# Molecular and antigenic characterization of *Trypanosoma cruzi* TolT proteins

**DOI:** 10.1371/journal.pntd.0007245

**Published:** 2019-03-14

**Authors:** Maite Lobo, Virginia Balouz, Luciano Melli, Giannina Carlevaro, María E. Cortina, María de los Milagros Cámara, Gaspar E. Cánepa, Santiago J. Carmona, Jaime Altcheh, Oscar Campetella, Andrés E. Ciocchini, Fernán Agüero, Juan Mucci, Carlos A. Buscaglia

**Affiliations:** 1 Instituto de Investigaciones Biotecnológicas “Dr Rodolfo Ugalde” (IIB-INTECh, Universidad Nacional de San Martín and CONICET), Buenos Aires, Argentina; 2 Servicio de Parasitología-Chagas, Hospital de Niños Ricardo Gutiérrez, Buenos Aires, Argentina; Instituto de Ciências Biológicas, Universidade Federal de Minas Gerais, BRAZIL

## Abstract

**Background:**

TolT was originally described as a *Trypanosoma cruzi* molecule that accumulated on the trypomastigote flagellum bearing similarity to bacterial TolA colicins receptors. Preliminary biochemical studies indicated that TolT resolved in SDS-PAGE as ~3–5 different bands with sizes between 34 and 45 kDa, and that this heterogeneity could be ascribed to differences in polypeptide glycosylation. However, the recurrent identification of TolT-deduced peptides, and variations thereof, in trypomastigote proteomic surveys suggested an intrinsic TolT complexity, and prompted us to undertake a thorough reassessment of this antigen.

**Methods/Principle findings:**

Genome mining exercises showed that TolT constitutes a larger-than-expected family of genes, with at least 12 polymorphic members in the *T*. *cruzi* CL Brener reference strain and homologs in different trypanosomes. According to structural features, TolT deduced proteins could be split into three robust groups, termed TolT-A, TolT-B, and TolT-C, all of them showing marginal sequence similarity to bacterial TolA proteins and canonical signatures of surface localization/membrane association, most of which were herein experimentally validated. Further biochemical and microscopy-based characterizations indicated that this grouping may have a functional correlate, as TolT-A, TolT-B and TolT-C molecules showed differences in their expression profile, sub-cellular distribution, post-translational modification(s) and antigenic structure. We finally used a recently developed fluorescence magnetic beads immunoassay to validate a recombinant protein spanning the central and mature region of a TolT-B deduced molecule for Chagas disease serodiagnosis.

**Conclusion/Significance:**

This study unveiled an unexpected genetic and biochemical complexity within the TolT family, which could be exploited for the development of novel *T*. *cruzi* biomarkers with diagnostic/therapeutic applications.

## Author summary

Chagas disease, caused by the protozoan *Trypanosoma cruzi*, is a lifelong and debilitating neglected illness of major significance in Latin America, for which no vaccine or adequate drugs are yet available. Identification of novel biomarkers able to transcend the current limits of diagnostic and/or therapeutic assessment methods hence surfaces as a main priority in Chagas disease applied research. In this framework, we herein undertook a thorough biochemical and antigenic characterization of *T*. *cruzi* TolT surface antigens. Our results unveil an unexpected complexity within this family, with at least 12 polymorphic TolT genes in the *T*. *cruzi* CL Brener reference strain genome. According to structural features, TolT deduced molecules could be split into three robust groups that show differences in their structural features, expression profile, sub-cellular distribution, post-translational modification(s) and antigenic structure. Overall, we show that TolT molecules are conspicuously expressed by both major mammal-dwelling stages of the parasite, and that they are differentially recognized by the immune system in Chagasic patients and in *T*. *cruzi*-infected mammals. Our findings are discussed in terms of the evolution and possible structural/functional roles of TolT molecules, as well as in terms of their applicability in Chagas disease serodiagnosis.

## Introduction

With ~6 million people already infected and ~100 million at risk of infection, Chagas disease constitutes the most important parasitic disease and leading cause of infectious cardiomyopathy in Latin America [1]. Migratory trends of infected populations from endemic areas to Europe, North America, and the Western Pacific have also led to the spreading of this illness, which is now recognized as an emerging threat to global public health [2]. *Trypanosoma cruzi*, the etiological agent of Chagas disease, is a protozoan parasite that transitions between vertebrates (including humans) and blood-sucking triatomine vectors, with different developmental stages involved in each host. Within the insect, two major developmental forms can be observed: replicative epimastigotes in the midgut and metacyclic trypomastigotes in the hindgut [3]. The latter forms bring the infection into mammals when deposited on the skin or mucosa along with the excreta of the bug during blood-feed. Following cell invasion, parasites differentiate into rounded amastigote forms [3]. Along this transformation, the parasite undergoes remarkable physiological and morphological changes [4], including the complete disposal of its flagellum [5]. After several rounds of replication and just before disruption of the parasite-laden cell, amastigotes differentiate back into non-dividing and highly motile bloodstream trypomastigotes, which disseminate the infection within the mammal and may be eventually taken up by the triatomine during a bloodmeal.

Following a 30–60 day-long acute phase, strong and parasite-specific immunity is elicited in *T*. *cruzi*-infected people [6]. However, the parasite ability to quickly invade a wide variety of cell types, and the concurrent deployment of multiple elaborated evasion systems turn this immune response only partially effective [6,7]. In this context, the surface coat of bloodstream trypomastigotes fulfills a key dual purpose: to interact with host cell receptors prior to parasite internalization, and to provide protection against mammalian host-derived defense mechanisms [7]. This coat is composed of densely packed glycosylphosphatidyl inositol (GPI)-anchored glycoconjugates, which are usually coded by large, polymorphic, and developmentally regulated gene families [8]. In quantitative terms, the most important trypomastigote coat glycoproteins are mucins, Gp85/*trans*-sialidases (TS) and mucin-associated surface proteins (MASPs), all of which distribute over the entire parasite cell body, the flagellum, and even the flagellar pocket [8].

In 1990, a novel type of *T*. *cruzi* trypomastigote antigen was identified [9]. This antigen turned out to display homology to bacterial TolA proteins [10], and was accordingly designated TolT (TolA-like protein from *T*. *cruzi*). Instead of showing a broad surface distribution, TolT localized exclusively to the trypomastigote flagellum [9], apparently in the part of this structure in contact with the parasite body. Western blot analysis showed that TolT actually consisted of ~3–5 different molecules with sizes between 34 and 45 kDa [9]. All of them however collapsed to a single species upon treatment with endoglycosidase H, suggesting they corresponded to identical and/or highly similar polypeptides undergoing differential glycosylation. Subsequent immunological screenings led to the identification of three genes (termed *TolT 1–3*) in the *T*. *cruzi* Esmeraldo strain, which were arranged in tandem following a head-to-tail disposition [11]. Two of these genes, *TolT1* and *TolT2* were identical at the nucleotide level, and showed 98.9% sequence identity with respect to *TolT3* [11]. The recurrent identification of peptides showing slight variations to *TolT 1–3* deduced sequences in recent proteomic surveys however hinted at an underestimated TolT complexity [12–16].

The complete DNA sequence of the *T*. *cruzi* CL Brener reference clone was released in 2005, and it is represented by two datasets of contigs, each corresponding to one parental haplotype, which are referred to as ‘Esmeraldo-like’ or ‘non-Esmeraldo-like’ [17]. The CL Brener genome revealed a highly repetitive structure, which corresponded to a marked expansion of transposable elements, satellite DNA, and large multigene families including the above mentioned mucins, TS and MASPs, usually organized in tandems [17]. These features, together with CL Brener hybrid nature resulted in a highly fragmented genome assembly [17]. The CL Brener genome was subsequently followed by that of distinct parasite strains/clones [18,19], and by the genomes of phylogenetically related organisms [20–23]. More recently, third-generation sequencing technologies and bioinformatics allowed high-quality genome assembly of *T*. *cruzi* genomes [24–26]. This wealth of genetic information, along with the pressing need of novel *T*. *cruzi* biomarkers [27], prompted us to revisit TolT.

In this work, we show that TolT constitutes a larger-than-expected family of genes in *T*. *cruzi*, with at least 12 polymorphic members in the CL Brener reference strain and homologs in different trypanosomes. According to structural features, TolT deduced proteins could be split into three robust groups, all of them showing homology to bacterial TolA proteins, a biased amino acid composition, canonical signatures of surface localization and/or secretion, and trypomastigote flagellar surface localization. All of them were also found to be expressed in amastigote forms, with a TolT group-specific sub-cellular distribution. Thorough biochemical and immunological characterizations indicated that distinct TolT groups show additional differences in their post-translational modification(s) and antigenic structure. We finally used a recently developed fluorescence magnetic beads immunoassay to validate a recombinant TolT-B protein as an appealing reagent for Chagas disease serodiagnosis.

## Materials and methods

### *In silico* predictions and phylogeny analyses

DNA sequences were compared using BLAST tool at the NCBI non-redundant DNA sequences databases at TriTrypDB (http://tritrypdb.org/tritrypdb/) and GeneDB (http://www.genedb.org/) using *TolT1* (GeneBank accession number AF099099) sequence as query. Sequences showing an *E* value < 10^−5^ (~40% identity) were retrieved and their complete open reading frames (ORFs) were aligned using T-Coffee. After manual curation of the output, a preliminary phylogenetic tree was built using the Neighbor-Joining method. This tree allowed the definition of 3 robust groups, termed TolT-A, TolT-B and TolT-C. The complete ORF of one representative member of each group (TolT-A: TcCLB.508767.20, TolT-B: TcCLB.510433.20, TolT-C: TcCLB.506815.20) was further used to perform ‘iterative’ screenings, using the same conditions as stated above. The final phylogram (made upon DNA sequences) is the consensus tree of 1,000 bootstrap replicates and was graphically modified for presentation using iTOL. In addition, the deduced polypeptide of each representative member was used to search for similar sequences in the protein databases at TriTrypDB and GeneDB. Identification of signal peptides (SP) and GPI-anchoring signals was done using the online servers SignalP 4.0 and PredGPI, respectively. Post-translational modifications were predicted using NetPhos 3.1, NetNGlyc 1.0, NetOGlyc 4.0 and CSS-Palm 3.0. Homology to TolA was evaluated by independently querying the bacterial database of UniProt (http://www.uniprot.org/blast/) under default conditions with each predicted TolT product. Logos were generated using WebLogo (http://weblogo.berkeley.edu/logo.cgi).

### Parasite stocks and cell lines

CL Brener developmental forms were obtained and purified as described [28]. Briefly, epimastigotes were grown at 28°C in brain-heart tryptose medium supplemented with 10% (v/v) heat-inactivated fetal calf serum (FCS). Cell-derived trypomastigotes (henceforth trypomastigotes) and extracellular amastigotes were harvested from the supernatant of Vero cells (ATCC) grown at 37°C and 5% CO_2_ in minimal essential medium (MEM) supplemented with 10% (v/v) FCS, 0.292 g/L *L*-glutamine, 100 IU/mL Penicillin and 100 μg/mL Streptomycin (all from GIBCO Laboratories).

### DNA extraction and gene amplifications

*T*. *cruzi* genomic DNA from CL Brener epimastigotes was purified as described [29]. Gene amplifications were obtained by PCR using 1–10 ng of DNA as template, recombinant Taq DNA Polymerase (Invitrogen), and the oligonucleotides detailed in S1 Table.

### RNA extraction, cDNA preparation and Real-time quantitative PCR (RT-qPCR)

Different parasite developmental forms (4 x 10^8^ of each) were homogenized in 1 mL of TRIzol reagent (Thermo), treated with DNAse I (Sigma), further partitioned in chloroform and centrifuged at 12,000 *x g*. The aqueous phase was recovered and RNA integrity was evaluated by 1% agarose gel electrophoresis. RNA was precipitated with 1 mL of 2-propanol. First strand cDNA was synthesized from total RNA samples using Superscript II reverse transcriptase (Life Technologies). Briefly, total RNA was resuspended in RNAse-free H_2_O and used at a final concentration of 0.25 μg/μL (3 μg of RNA per reaction), 10 μM oligo-dT-anchor primer (S1 Table), and 10 mM dNTPs in the reverse transcriptase (RT) First Strand Synthesis kit (Sigma). RT reactions were diluted appropriately, and used as templates for Real-Time quantitative PCR (RT-qPCR) reactions using Kapa Sybr Fast Universal Kit (Biosystems) and primers (S1 Table) were designed using PerlPrimer software v1.1.21. To verify that the SYBR Green dye detected only one PCR product, all the reactions were subjected to the heat dissociation protocol after the final cycle of the PCR and sequenced [30]. Samples were tested against *T*. *cruzi* Calmodulin and Glyceraldehyde 3-phosphate dehydrogenase (TcGAPDH) as reference genes for data normalization (S1 Table). Each experiment was performed in triplicate for two independently generated sets of cDNA templates.

### Cloning procedures

PCR amplicons corresponding to TolT molecules were cloned into the pGEM-T easy vector (Promega), and used to transform DH5α cells (Invitrogen). DNA sequencing was carried out at Macrogen. These amplicons were then digested with the indicated restriction enzymes (S1 Table) and cloned into a tailored version of pGEX-2T (GE Healthcare) [31]. The glutathione *S*-transferase (GST)-fusion protein bearing the repetitive domain of *T*. *cruzi* antigen 1 (Ag1, also known as FRA, JL7 or H49 [27]) has been described [32].

### Expression of recombinant proteins in bacteria and antibody development

Soluble fractions of *E*. *coli* strain BL21-Codon Plus (DE3)-RP cultures induced for 3 h at 28°C with 0.1 mM isopropyl ß-D-thiogalactopyranoside (Fermentas) were purified by gluthatione-Sepharose chromatography (GE Healthcare) and dialyzed against PBS [32,33]. GST, GST-Ag1, and GST-TolT samples were quantified by Bradford reagent (Bio-Rad) and purity was assessed by Coomasie blue-stained SDS-PAGE. Purified GST-TolT proteins were injected into animals as described [34] to generate specific antisera (S2 Table). Antiserum to *T*. *cruzi* TSSA has been described [35].

### Indirect immunofluorescence (IIF) assays

For IIF assays, trypomastigote forms (~10^6^) were harvested, washed in PBS, adhered to poly-*L*-lysine (Sigma)-coated cover-slips and fixed for 30 min in PBS containing 4% (v/v) *p*-formaldehyde (PBS-PFA). Parasites were blocked for 30 min in 5% (w/v) Bovine Serum Albumin (Sigma) in PBS (PBS-A) supplemented with 0.5% (w/v) saponin (Sigma) for permeabilization, and probed with the indicated antiserum diluted in PBS-A. After extensive washings with PBS-A, secondary Alexa Fluor-conjugated antibodies (Molecular Probes) were added. Nuclei were stained with DAPI prior to montage in FluorSave reagent (CalBiochem). To evaluate the reactivity of *T*. *cruzi* intracellular stages 10,000 Vero cells were plated onto round coverslips, let stand overnight and infected with 1 x 10^6^ CL Brener trypomastigotes per coverslip as described [35]. After 5 h, trypomastigotes were removed and cells were extensively washed and incubated in DMEM 10% (v/v) SFB. At 72–120 h post-infection, cells were washed with PBS, fixed and processed for IIF as above. Images were obtained with a Nikon Eclipse 80i epi-fluorescence microscope coupled to a DS-Qi1 CCD camera, and processed using ImageJ. For co-localization analyses, trypomastigotes fixed and adhered to cover-slips as above were blocked with PBS supplemented with 3% (w/v) BSA and 2% (v/v) horse serum (PBS-AHS) and incubated with rat anti-TolT-A and mouse anti-TolT-B sera (both diluted 1:100 in PBS-AHS). Following extensive washes, secondary antibodies were added for 1 h at 1:1,000 dilution in PBS-AHS. Images were obtained with an Olympus IX-81 microscope attached with a FV-1000 confocal module. Co-localization analysis was assessed using the Co-localization Finder plugin from ImageJ.

### Immunoprecipitation

CL Brener trypomastigotes (2 x 10^8^) were resuspended in 1 ml of ip buffer (PBS supplemented with 0.1% (v/v) Triton X-100 and a protease inhibitor cocktail (Sigma-Aldrich)). The extract (input fraction) was added with 6 μl of TolT-A antiserum raised in rat and incubated overnight at 4°C under constant agitation. This mixture was then added with 150 μl of protein G-Sepharose (GE Healthcare) previously washed with 500 μl of ip buffer, and incubated for 3 h at 4°C under constant agitation. The tube was centrifuged at 600 *x g* for 1 min. The supernatant (flow-through fraction) was removed, resin washed five times in 1 ml each of ip buffer, and stripped at 100°C for 5 min in SDS-PAGE loading buffer: 50 mM Tris-HCl pH6.8, 1% (w/v) SDS, 0.01% (w/v) bromophenol blue, 10% (v/v) glycerol and 50 mM dithiothreitol (DTT).

### Gel electrophoresis and Western blots

To achieve non-reducing conditions, the reducing reagent (DTT) was omitted from the SDS-PAGE loading buffer (see composition above). Samples were mixed with the indicated loading buffer and heated at 100°C for 3 min before being processed for Western blot as described [14]. Briefly, extracts from 1.5 x 10^7^ parasites were resolved into SDS-PAGE (12.5% gels), transferred onto PVDF membranes (GE Healthcare), reacted with the indicated antiserum followed by HRP-conjugated secondary antibodies and developed using enhanced chemiluminescence (Thermo).

### Purification of GPI-anchored proteins and phosphoinositol specific phospholipase C (PI-PLC) treatment

Pellets containing 1–5 x 10^8^ trypomastigotes were homogenized in 2 mL of GPI buffer [10 mM Tris/HCl, pH 7.4, 150 mM NaCl, 2% (v/v) Triton X-114, 1 mM PMSF and 1% (v/v) protease inhibitor cocktail (Sigma)] on ice for 1 h and centrifuged at 8,800 *x g* for 10 min at 0°C as described [36]. The supernatant (S1) was stored at −20°C for 24 h. The pellet (P1) was washed with 1 ml of buffer A (10 mM Tris/HCl, pH 7.4, 150 mM NaCl, 0.06% (v/v) Triton X-114 and 1 mM PMSF) and stored. S1 was thawed and submitted to phase separation at 37°C for 10 min followed by centrifugation at 3,000 x *g* for 3 min at room temperature. The upper phase (S2) was collected and the detergent-rich phase was re-extracted with 1 ml of buffer A. The upper phase (S3) was collected, and the detergent rich phase was extracted with 1 ml of buffer A, homogenized, incubated for 30 min at 0°C and centrifuged at 18,000 x *g* for 10 min at 0°C. The pellet (P2) was washed with 1 ml of buffer A and stored, whereas the supernatant (S4) was submitted to a new phase separation. The upper phase (S5) was collected and the lower detergent-rich phase, enriched in GPI-anchored proteins, was taken as the GPI fraction (GPI). For PI-PLC treatment, 5 x 10^7^ trypomastigotes were treated with or without 0.1U of recombinant PI-PLC from *Bacillus cereus* (Thermo) for 30 min at 4°C. Normal morphology and motility was controlled by microscopic observation before and after the incubation time. Following PI-PLC treatment, parasite pellets and supernatants were separated by centrifugation and fractions were processed for Western blot.

### Concanavalin A fractionation and endoglycosidase H treatment

Trypomastigote pellets (3 x 10^8^) were homogenized in 500 μL of ConA buffer [50 mM Tris/HCl, pH 7.4, 150 mM NaCl, 1% (v/v) Triton X-100, 0.1% (v/v) Nonidet P40, 0.1% (w/v) sodium deoxycholate, 5 mM Cl_2_Ca, 5 mM Cl_2_Mg, 5 mM Cl_2_Mn, 1% (v/v) inhibitor protease cocktail (Sigma), and 1 mM DTT] and processed as described [36]. After clarification, parasite extract was fractionated overnight at 4°C onto 100 μL of ConA–Sepharose (GE Healthcare), and retained glycoproteins eluted with 50 μL of SDS-PAGE loading buffer. Flow-through and ConA-bound fractions were analyzed by Western blot with different antisera. For *N*-glycosylation analysis, trypomastigotes were lysed in 1X Glycoprotein Denaturing Buffer (BioLabs), boiled for 10 min and extracts corresponding to 2.5 x 10^7^ parasites were treated with 2,000–3,000U of Endoglycosidase H for 1 h following manufacturer’s procedures (BioLabs) and analyzed by Western blot.

### Peptide microarrays

Synthesis, screening and data analysis of high-density *T*. *cruzi*-derived peptide microarrays have been described [31,37].

### Enzyme-linked Immunosorbent Assay (ELISA)

GST-fusion proteins were dissolved in carbonate buffer (pH 9.6) at 10 μg/mL. Flat-bottomed 96-well Nunc-Immuno plates (Nunc, Roskilde, Denmark) were coated overnight at 4°C with 100 μL of the antigen solution, washed 3 times with PBS containing 0.05% (v/v) Tween 20 (PBS/T), and blocked for 1 h with 4% (w/v) skim milk in PBS/T at 37°C [33]. The plates were washed 3 times with PBS/T prior to the addition of serum samples diluted 1:500 in blocking buffer. Following incubation for 1 h at 37°C and washings with PBS/T, HRP-conjugated goat IgG to species-specific IgG (all from Sigma) diluted 1:5,000 in blocking buffer was added to the plates, and incubated at 37°C for 1 h. The plates were next washed and incubated with 100 μL of freshly prepared 0.5 mM 3,3’,5,5’-tetramethylbenzidine (Sigma) in citrate-phosphate buffer (pH 4.2) containing 0.2% (v/v) hydrogen peroxide. The reaction was stopped with 50 μL of 2 M sulfuric acid, and the absorbance at 450 nm was read. Each sample was assayed in triplicate, unless otherwise indicated.

### Fluorescence magnetic beads immunoassays (FMBIA)

Synthesis and coating of superparamagnetic microbeads with purified GST-fusion proteins were performed as described [32]. Functionalized beads (0.5 μg of each antigen in 20 μL of beads per reaction) were incubated with human serum samples (1:100 dilution), washed three times and bound antibodies were detected with DyLight 650-conjugated goat anti-human IgG antibodies (1:1,000 dilution, Jackson ImmunoResearch Laboratories). After washing three times, fluorescence was directly determined using a plate fluorometer (DTX880 Multimode Detector, Beckman Coulter). Incubation of coated beads with serum samples and conjugate antibodies were carried out for 5 min each, at room temperature without agitation. Sample and conjugate antibody dilutions, as well as washes between incubation steps were performed with PBS containing 0.2% (v/v) Tween 20. Washes were done using a magnetic rack without the need of centrifugation. Results of the FMBIA were expressed as percentage of reactivity of the mean fluorescence units of a standardized, positive control serum included in each assay run [32].

### Study population

Serum samples from *T*. *cruzi*-infected subjects have been described [32,33,38], and were obtained from the Laboratorio de Enfermedad de Chagas, Hospital de Ninos "Dr. Ricardo Gutierrez". All procedures were approved by the research and teaching committee and the bioethics committee of this institution, and followed the Declaration of Helsinki Principles. Written informed consent was obtained from all individuals (or from their legal representatives), and all samples were decoded and de-identified before they were provided for research purposes. Chagasic patients were coursing the chronic stage of the disease without cardiac or gastrointestinal compromise. Serum samples were analyzed for *T*. *cruzi*-specific antibodies with the following commercially available kits: ELISA using total parasite homogenate (Wiener lab, Argentina) and indirect hemmaglutination assay (IHA, Polychaco, Argentina). The participating subjects currently live within the urban limits of Buenos Aires, an area free of vector-borne parasite transmission, though they (or their parents) were born and raised in endemic areas from Argentina or neighbor countries, where most likely acquired *T*. *cruzi* infection. Serum samples from healthy individuals that gave negative results in the aforementioned tests were obtained from different blood banks: Fundación Hemocentro Buenos Aires (Buenos Aires, Argentina), Hospital de Enfermedades Infecciosas ‘Dr. Francisco Javier Muñiz’ (Buenos Aires, Argentina), Hospital Italiano de Buenos Aires (Buenos Aires, Argentina) and Hospital Municipal ‘Dr. Diego E. Thompson’ (San Martín, Buenos Aires, Argentina).

### Ethics statement

The Institutional Review Board of UNSAM has evaluated the current project and considered that it complies with the Basic HHS Policy for Protection of Human Research Subjects requirements to be included in the ‘exemption 4', because it involved the use of de-coded and de-identified human serum samples obtained from sera repositories. The protocol of animal immunization followed in this study was approved by the Committee on the Ethics of Animal Experiments of the Universidad Nacional de San Martín (IACUC/CICUAE N° 08/2015), and all the procedures were carried out according with the recommendations of the Guide for the Care and Use of Laboratory Animals of the National Institutes of Health.

## Results

### Identification and in silico characterization of TolT genes in trypanosomatids

**Fig 1:**
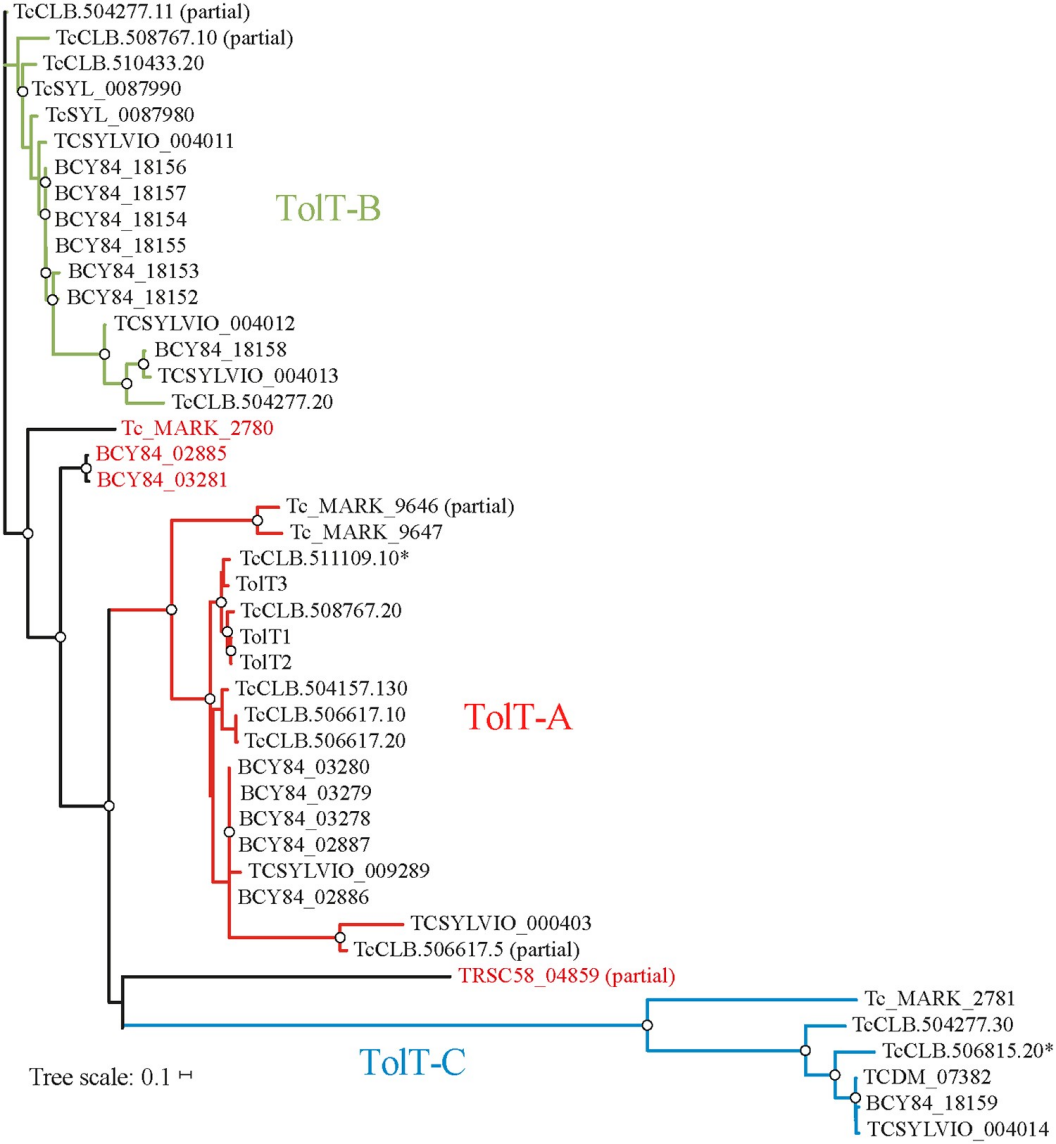
Evolutionary relationship of *TolT* genes in trypanosomatids. An unrooted phylogenetic tree was constructed from nucleotide alignments using the Neighbor-Joining method and bootstrapped using 1,000 permutations. Bootstrap support values > 70 are indicated by dots. The scale indicates the nucleotide substitution distance along the branches. Sections of the tree are colorized according to the TolT group to which they belong. Genes that cannot be included in any cluster and pseudogenes are indicated with red letters and asterisks, respectively. Abbreviations: TcCLB, *T*. *cruzi* CL Brener; TcSYL or TcSYLVIO, *T*. *cruzi* Sylvio X-10; TCDM or BCY84, *T*. *cruzi* Dm28c; Tc_MARK, *T*. *cruzi marinkellei*; TRSC, *Trypanosoma rangeli* SC58.

Searches in the kinetoplastid genomic databases at TriTrypDB and GeneDB were carried out using the complete ORF of *T*. *cruzi TolT1* as bait [11]. A total of 43 different sequences showing significant similarity (> 40% nucleotide identity) to *TolT1* were retrieved; 38 sequences from different isolates of *T*. *cruzi* (CL Brener, Sylvio X-10, Dm28c, Esmeraldo) and 5 sequences from phylogenetically related protozoa such as the bat parasite *T*. *cruzi marinkellei* (4 sequences) and the human parasite *Trypanosoma rangeli* (1 sequence). Further searches were carried out using different TolT deduced protein sequences as query, which allowed for the identification of an additional TolT molecule in the reptilian parasite *Trypanosoma grayi* (DQ04_05721021). No TolT-related sequence was found in the genus *Leishmania* or in strict salivarian trypanosomes, i.e. trypanosomes that develop in the salivary glands of the insect vector such as *Trypanosoma brucei*, *Trypanosoma congolense*, *Trypanosoma evansi* and *Trypanosoma vivax*. A phylogenetic tree based on Neighbor-Joining method allowed the definition of 3 main groups of TolT-related sequences (termed TolT-A, -B and -C), which were supported by significant bootstrap values (Fig 1).

**Fig 2:**
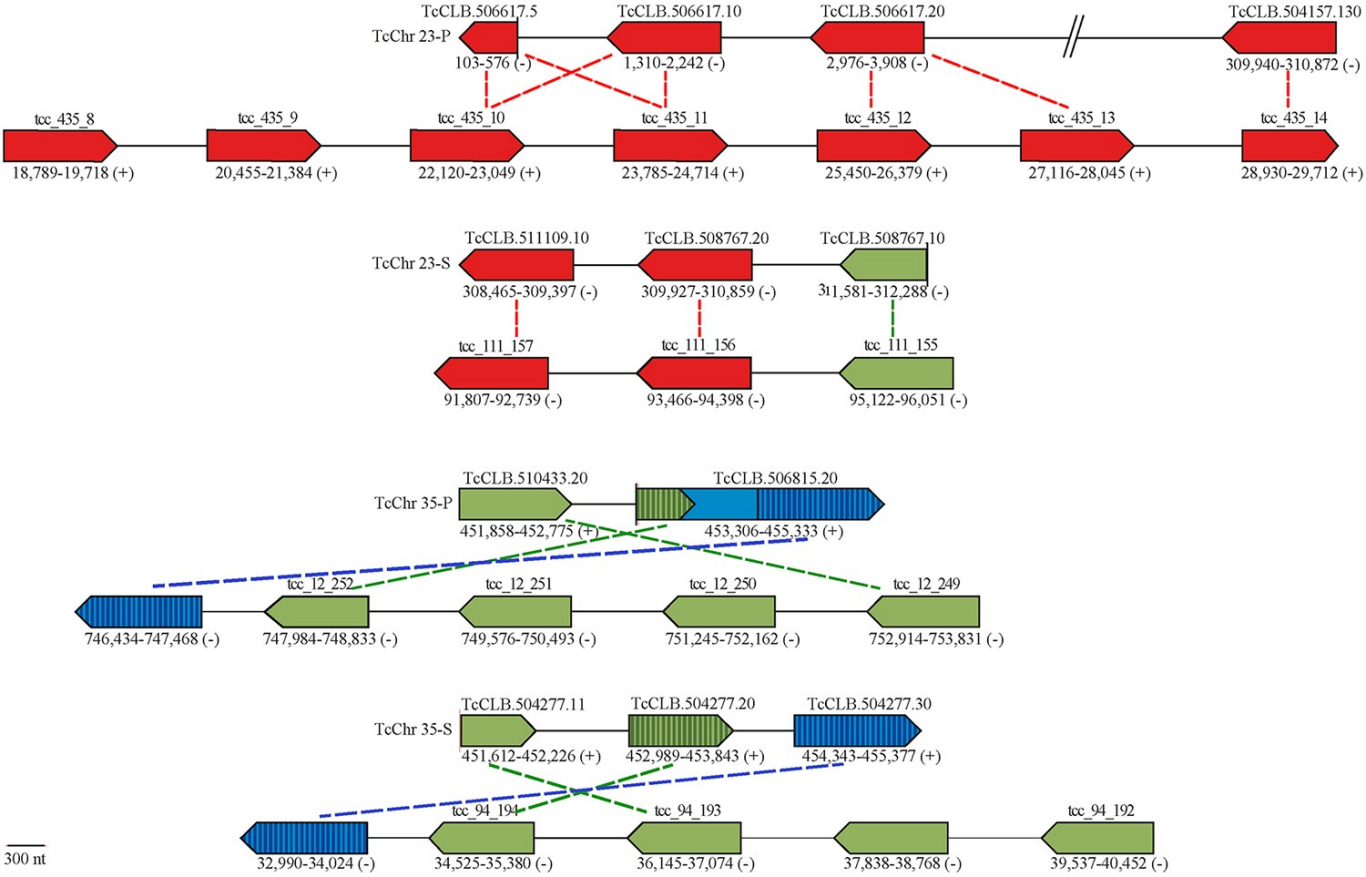
Genomic organization of *TolT* genes in *T*. *cruzi* CL Brener and TCC strains. Schematic representation of *T*. *cruzi* CL Brener (above) and TCC (below) genomic scaffolds containing *TolT* sequences. Vertical lines denote contig breaks in the CL Brener genome, and green and blue striped regions in TcCLB.506815.20 indicate sequences bearing high similarity to TcCLB.504277.20 and TcCLB.504277.30, respectively. Orthologous sequences (100% nucleotide identity) are connected by dotted lines. Abbreviations: TcChr-S and -P, ‘Esmeraldo’ and ‘non-Esmeraldo’ haplotype, respectively, of the hybrid CL Brener clone. Genes are colorized according to the TolT group to which they belong, as defined in Fig 1.

TolT-A sequences showed ~96–100% identity to *TolT1* and included the 3 original sequences from the *T*. *cruzi* Esmeraldo strain [11], 5 full-length sequences from the *T*. *cruzi* CL Brener clone (TcCLB.508767.20, TcCLB.506617.10, TcCLB.506617.20, TcCLB.504157.130 and TcCLB.511109.10) and one partial sequence, also from CL Brener (TcCLB.506617.5) bearing *C*-terminal truncation (Fig 1). As originally described in the Esmeraldo strain [11], TolT-A sequences in CL Brener were arranged in a head-to-tail assembly of 3 members, which mapped to chromosome 23 (Fig 2). A fourth gene, TcCLB.504157.130, could not be linked to this cluster most likely due to CL Brener genome assembly deficiencies. Similar analyses performed on *T*. *cruzi* TCC, a hybrid strain from the same evolutionary lineage than CL Brener with a high-quality genome assembly [26], strongly supported the inclusion of TcCLB.504157.130 within the CL Brener TolT-A cluster found in chromosome 23. Moreover, they suggested the existence of additional TolT-A members in such CL Brener cluster that may have collapsed during genome assembly (Fig 2). TcCLB.511109.10 was annotated as a pseudogene due to a single nucleotide deletion that originated a frame shift mutation within the deduced *N*-terminal signal peptide (S1 Fig). Downstream from this mutation, however, TcCLB.511109.10 sequence was > 96% identical to the remaining TolT-A genes (S1 Fig), suggesting that it constitutes either a very recent pseudogene or, most likely, a sequencing error. Indeed, the orthologous TCC gene (tcc_111_157, Fig 2) was 100% identical to TcCLB.511109.10 except for this single nucleotide deletion. The TolT-A group also included 5 sequences from *T*. *cruzi* Dm28c, 1 from *T*. *cruzi* Silvio X-10 and 2 from *T*. *cruzi marinkellei* (Fig 1).

The TolT-B group displayed 76–90% identity to *TolT1* and comprised 2 full-length sequences (TcCLB.510433.20 and TcCLB.504277.20) and 2 partial sequences bearing *N*-terminal truncations (TcCLB.504277.11 and TcCLB.508767.10) in the CL Brener genome (Fig 2). TolT-B sequences mapped to a single cluster present in chromosome 35 except for TcCLB.508767.10, which localized immediately downstream to TcCLB.508767.20 within the ‘Esmeraldo’ haplotype of the TolT-A genomic cluster (Fig 2). The TCC genome supported this particular disposition and, again, suggested the existence of additional TolT-B genes in the CL Brener chromosome 35 cluster (Fig 2). TolT-B also included 5 sequences from *T*. *cruzi* Sylvio X-10 and 7 sequences from *T*. *cruzi* Dm28c (Fig 1). Interestingly, TcCLB.510433.20 and TcCLB.504277.20 were almost identical sequences except for their predicted *C*-termini, where they become highly divergent at both nucleotide and amino acid sequences (S2 Fig). Because of these differences, the TcCLB.504277.20 deduced protein was predicted to lose the GPI-anchoring signal. The identification of orthologous genes in Sylvio X-10 (TcSYL_004013) and TCC (tcc_94_104) strongly argued that ‘GPI-less’ variants are not the result of genome assembly problems but rather genuinely diversified TolT molecules (S2 Fig).

The TolT-C group was composed by just two alleles of a single gene (TcCLB.504277.30 and TcCLB.506815.20) within the CL Brener genome (Fig 2). This locus mapped immediately downstream of the TolT-B genomic cluster present in chromosome 35; and a quite similar disposition was also observed in TCC (Fig 2). TcCLB.506815.20 was annotated as a *MASP* pseudogene. However, manual inspection allowed us to identify a partial TolT-B gene, a putative intergenic region and a complete TolT-C gene within this sequence (Fig 2 and S3 Fig). The TolT-C group also included sequences retrieved from *T*. *cruzi* Dm28c, *T*. *cruzi* Sylvio X-10 and *T*. *cruzi marinkellei* (Fig 1).

### TolT proteins in T. cruzi

**Fig 3:**
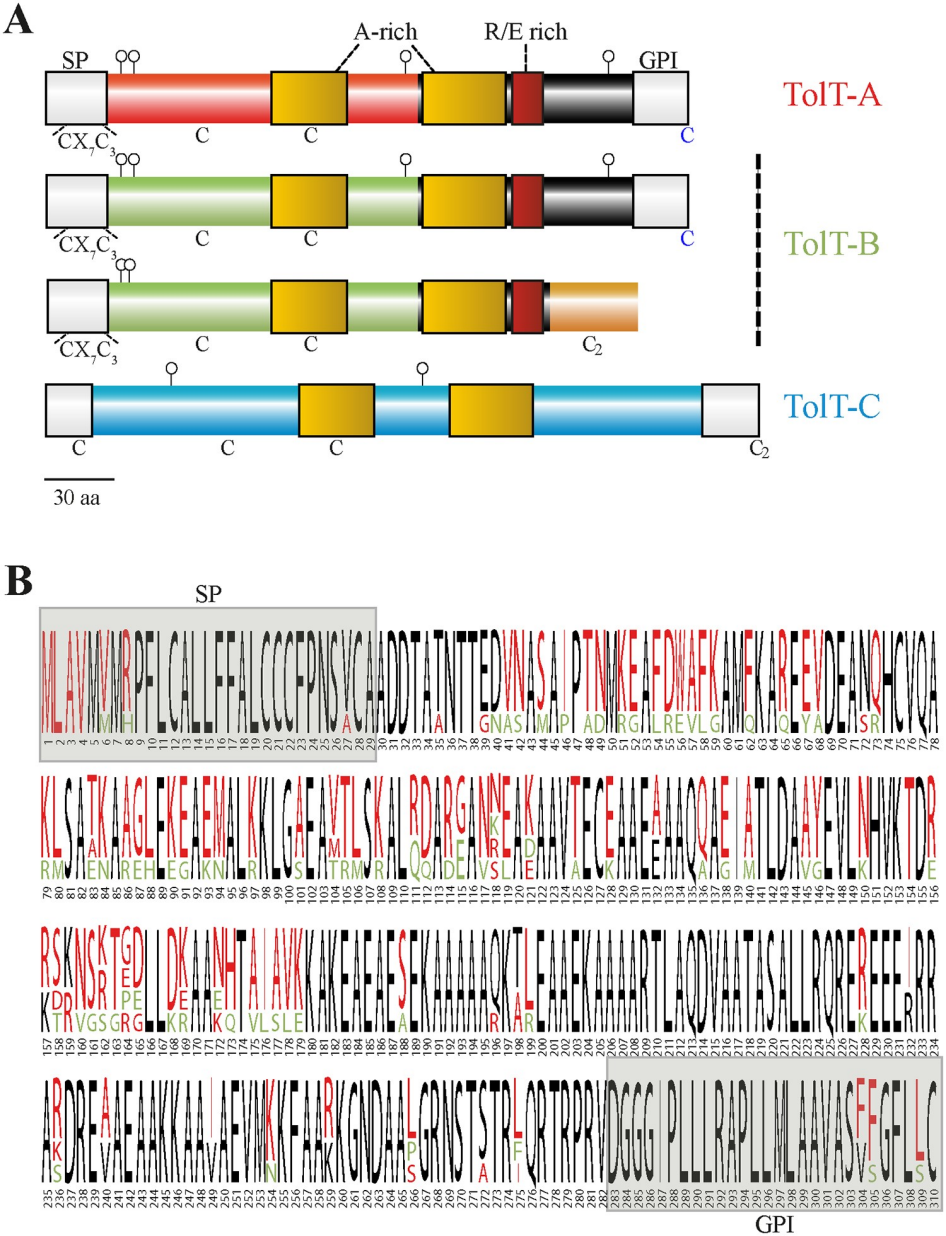
Diversity of TolT products in *T*. *cruzi*. **A)** Schematic illustration of predicted TolT products. For TolT-B, one ‘canonical’ and one ‘GPI-less’ variants are shown. The predicted signal peptide (SP) and GPI-anchoring sequences (GPI) are indicated in grey. Alanine (A)-rich domains and the arginine (R)/glutamic acid (E)-rich motif are denoted as yellow and red boxes, respectively. *N*-glycosylation sequons are indicated as pins. Conserved cysteine residues are shown, and those predicted to undergo palmitoylation are colored blue. **B)** Multiple alignments results of TolT-A (TcCLB.508767.20, TcCLB.506617.10, TcCLB.506617.20 and TcCLB.504157.130) and ‘canonical’ TolT-B (TcCLB.504277.20 and TcCLB.510433.20) protein sequences are depicted as *WebLogo* graphics. Residues from TolT-A or TolT-B sequences are indicated by red and green letters, respectively, whereas residues conserved between both groups are indicated in black letters.

The main proteins encoded by TolT-A, TolT-B and TolT-C bore 310, 305/284 and 344 amino acids, respectively, with predicted molecular masses of ~30.9–37.1 kDa and predicted p*I* of 7.1–8.2. All of them displayed sequences that constitute canonical signatures of surface localization and/or secretion, including a predicted *N*-terminal signal peptide (SP) and a *C*-terminal GPI-anchoring signal (except for the ‘GPI-less’ TcCLB.504277.20) (Fig 3A). In addition, different algorithms predicted the existence of palmitoylation signals in TolT-A and TolT-B ‘canonical’ proteins (Fig 3A). The deduced TolT polypeptides were characterized by a high content of Ala (18.8 to 24.4%), Glu (9.0 to 11.9%), Leu (8.1 to 9.9%), Lys (6.3 to 10.4%) and Arg (4.9 to 8.1%), with these residues not evenly distributed throughout the entire proteins (Fig 3A). In addition, TolT-A and TolT-B products displayed a Cys-X_7_-Cys_3_ motif (where X means any residue) in their predicted SP (Fig 3A). Interestingly, this motif is also present in the SP of *T*. *cruzi* mammal-dwelling-expressed mucins (TcMUC, [28,39]), suggesting that TcMUC and TolT molecules may have a common origin or, more likely, that this motif may have been selected for the improved expression and/or post-translational processing of surface-associated molecules in such parasite forms (see below).

The predicted mature TolT molecules, i.e. upon processing of the SP and, if present, the GPI-anchoring signal displayed 17–41 potential sites for phosphorylation, 2–5 sites for *N*-glycosylation, 27–36 sites for *O*-glycosylation, and 2 strictly conserved Cys residues (Fig 3A). The only recognizable and unifying feature was the presence of two Ala-rich regions, which are predicted to fold into an αhelix-enriched secondary structure (Fig 3A). These regions, along with the interconnecting sequence, displayed structural and marginal sequence similarity to the central domain of bacterial TolA proteins. TolT-A and TolT-B products, in addition, bore a particular region towards the mature *C*-terminus, which is highly enriched in Arg and Glu residues (Fig 3A).

Amino acid alignments highlighted very few and minor intra-group polymorphisms among CL Brener TolT proteins, the only exception being the *C*-terminus of the ‘GPI-less’ variant encoded by TcCLB.504277.20 (S4 Fig). When comparing between groups, the genetic drift of TolT-C proteins and the high level of sequence conservation among TolT-A and TolT-B members were also evident (S4 Fig). Regarding the latter issue, it is worth noting that the amino acid identity value between TolT-A and ‘canonical’ TolT-B deduced products was not homogeneous. Rather, and as schematized in Fig 3B, this value was maximal along their predicted SP and *C-*terminal region but dropped down significantly towards their predicted mature *N*-terminal region (from residues ~40 to 180).

### Expression of TolT RNAs and proteins during the T. cruzi life-cycle

**Fig 4:**
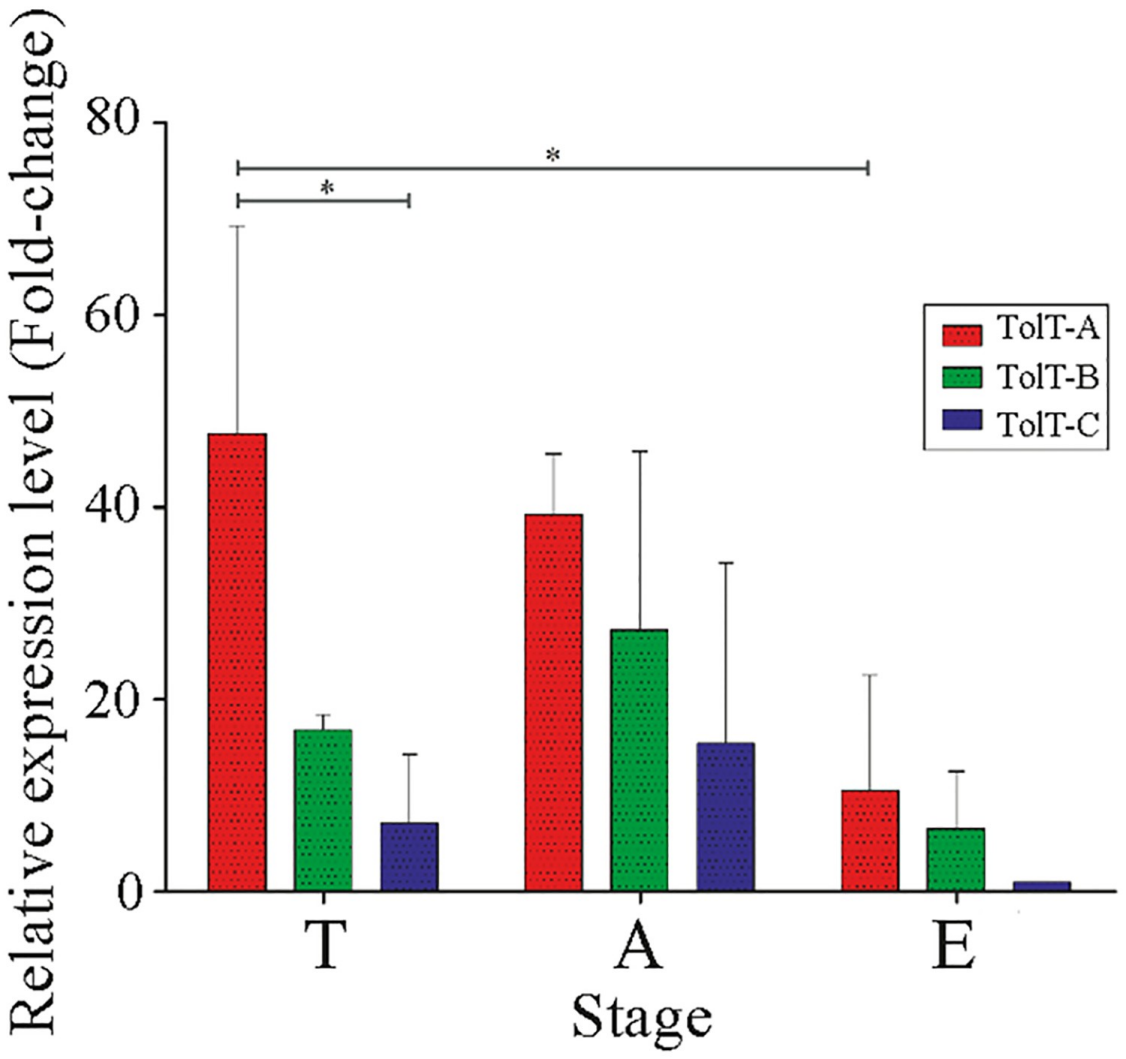
Expression analysis of TolT mRNAs in *T*. *cruzi*. mRNA expression profile of different TolT groups on major developmental stages of *T*. *cruzi* CL Brener. Asterisks denote significant differences between the population means (*P <* 0.05) assessed by two-way ANOVA test after Bonferroni’s correction. A, amastigote; T, trypomastigote; E, epimastigote.

Samples of total RNA from different *T*. *cruzi* CL Brener developmental forms were purified and the relative expression of representative members of each TolT group evaluated by RT-qPCR. As shown in Fig 4, TolT-A transcripts were the most abundant, followed by TolT-B and TolT-C. Abundance of *TolT* mRNAs was significantly decreased in epimastigotes, particularly for TolT-C, for which transcript expression was barely detectable (Fig 4). When comparing between mammal-dwelling stages, i.e. trypomastigotes vs amastigotes, and somehow at odds with early steady-state transcriptome analyses [40], no significant differences were observed for either TolT group (Fig 4).

**Fig 5:**
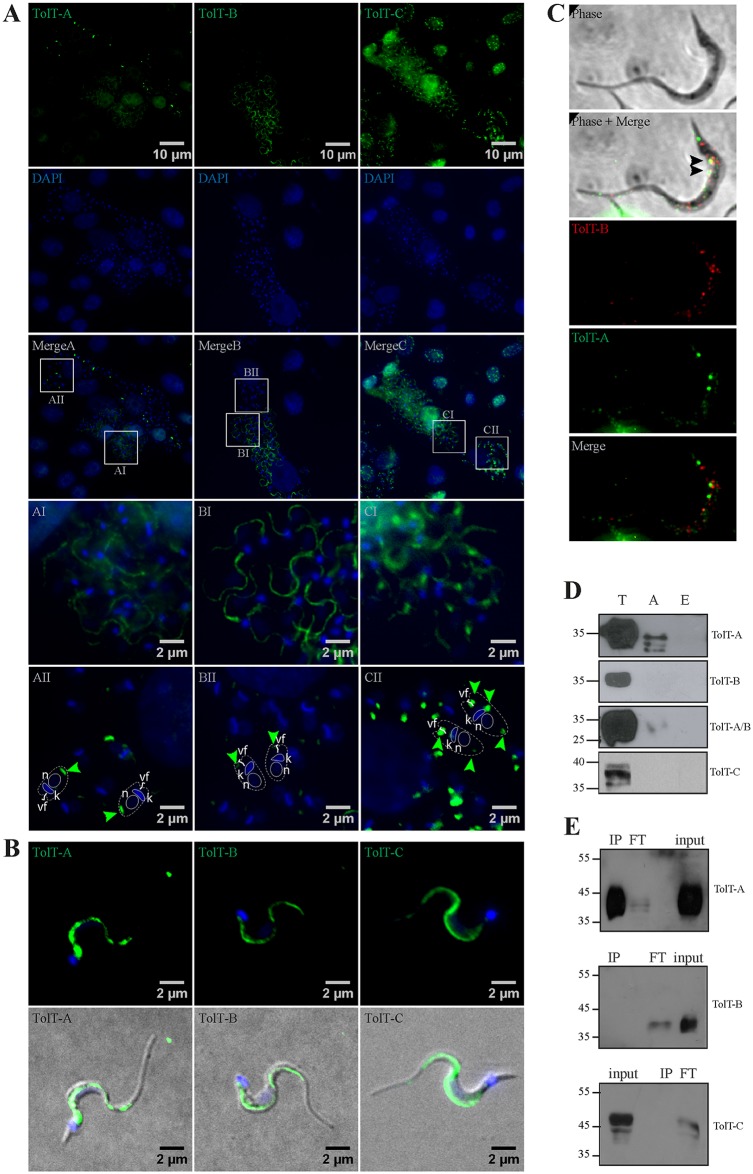
Expression analysis of TolT products in *T*. *cruzi*. **A** and **B)** *T*. *cruzi*-infected HeLa cells (A) or purified trypomastigotes (B) were permeabilized and analyzed by indirect immunofluorescence (IIF) assays using the indicated antiserum. DAPI signals are shown in blue. Enlarged regions are depicted with squares in the original merge images. In the lower panels, a schematic drawing of amastigote forms in which the nucleus (n), the kinetoplastid DNA (k) and the vestigial flagellum (vf) are indicated. Arrowheads point to observed TolT signals. **C)** Co-localization analyses. Representative confocal image of trypomastigote processed for IIF using rat TolT-A antiserum (in green) and mouse TolT-B antiserum (in red). Arrowheads point to co-localizing signals. **D)** Total extracts of different parasite stages (A, amastigote; T, trypomastigote; E, epimastigote) were probed with the indicated antiserum by Western blotting. Molecular markers (in kDa) are indicated. **E)** Total extracts of purified trypomastigotes were immunoprecipitated using rat TolT-A antiserum and input, flow-through (FT) and immunoprecipitated (IP) fractions were analyzed by Western blot using the indicated mouse antiserum. All of the parasites shown in this figure were from CL Brener strain.

We next produced antisera against recombinant, GST-fusion proteins spanning sequences of different TolT molecules (S2 Table). To minimize the extent of possible cross-recognition between TolT-A and TolT-B molecules, specific antisera were raised against sequences from their most divergent, mature *N*-terminal regions (Fig 3). We also generated an antiserum to the TolT-A and TolT-B conserved *C-*terminal region (henceforth TolTA/B antiserum, S2 Table). As for the most divergent TolT-C group, we generated an antiserum towards a GST-fusion protein spanning most of its central region (S2 Table). These antisera were used to determine expression of TolT products along the parasite life cycle. IIF assays on *T*. *cruzi*-infected cells indicated that TolT-A and particularly TolT-B variants were significantly more expressed in trypomastigotes as compared to amastigotes (Fig 5A). The TolT-C antiserum, on the other hand, yielded similar signals in both parasite stages (Fig 5A). TolT-C signals, in turn, were weaker than those recorded for TolT-A and TolT-B, and should be thus compensated for presentation.

As originally reported for ‘TolT’ [9], TolT-A, TolT-B and TolT-C molecules localized to the part of the flagellum in contact with the parasite body. This was verified in both intracellular and extracellular trypomastigotes (Fig 5A and 5B). It is however worth noting that TolT-C molecules displayed an apparent continuous distribution whereas TolT-A and TolT-B proteins yielded a more punctuated labeling pattern (Fig 5B). Confocal images strongly supported TolT-A and TolT-B discontinuous distribution (Fig 5C). Most importantly, they revealed only minor co-localization between TolT-A and TolT-B signals (Fig 5C; Pearson’s R correlation coefficient of co-localization = -0.3).

In addition to the trypomastigote flagellum, some TolT-A antisera (but not all of them) also labeled a discrete region towards the posterior end, i.e. the parasite pole opposed to the site of emergence of the (vestigial) flagellum of amastigote forms (Fig 5A). This was consistent with Western blot data, showing the presence of TolT-A-reactive species, though in significantly lesser amounts as compared to trypomastigote ones, in amastigote forms (Fig 5D). Interestingly amastigote- and trypomastigote-expressed TolT-A molecules displayed different electrophoretic mobility, suggesting differences in their post-translational processing (Fig 5D). TolT-B and TolT-C antisera also labeled discrete internal region(s) of amastigote forms by IIF assays (Fig 5A). At variance with TolT-A, TolT-B signals accumulated towards the anterior pole of the amastigote whereas TolT-C products accumulated at both amastigote tips, and also at a compartment juxtaposed to the kinetoplast DNA (kDNA, Fig 5A). Amastigote-expressed TolT-B and TolT-C species could not be detected by Western blot (Fig 5D), most likely due to differences in the sensitivity of both methods. Neither antiserum displayed specific reactivity towards epimastigote stages (Fig 5D). Immunoprecipitation assays further demonstrated that the generated antisera were TolT group specific (Fig 5E).

### Biochemical features of TolT proteins

**Fig 6:**
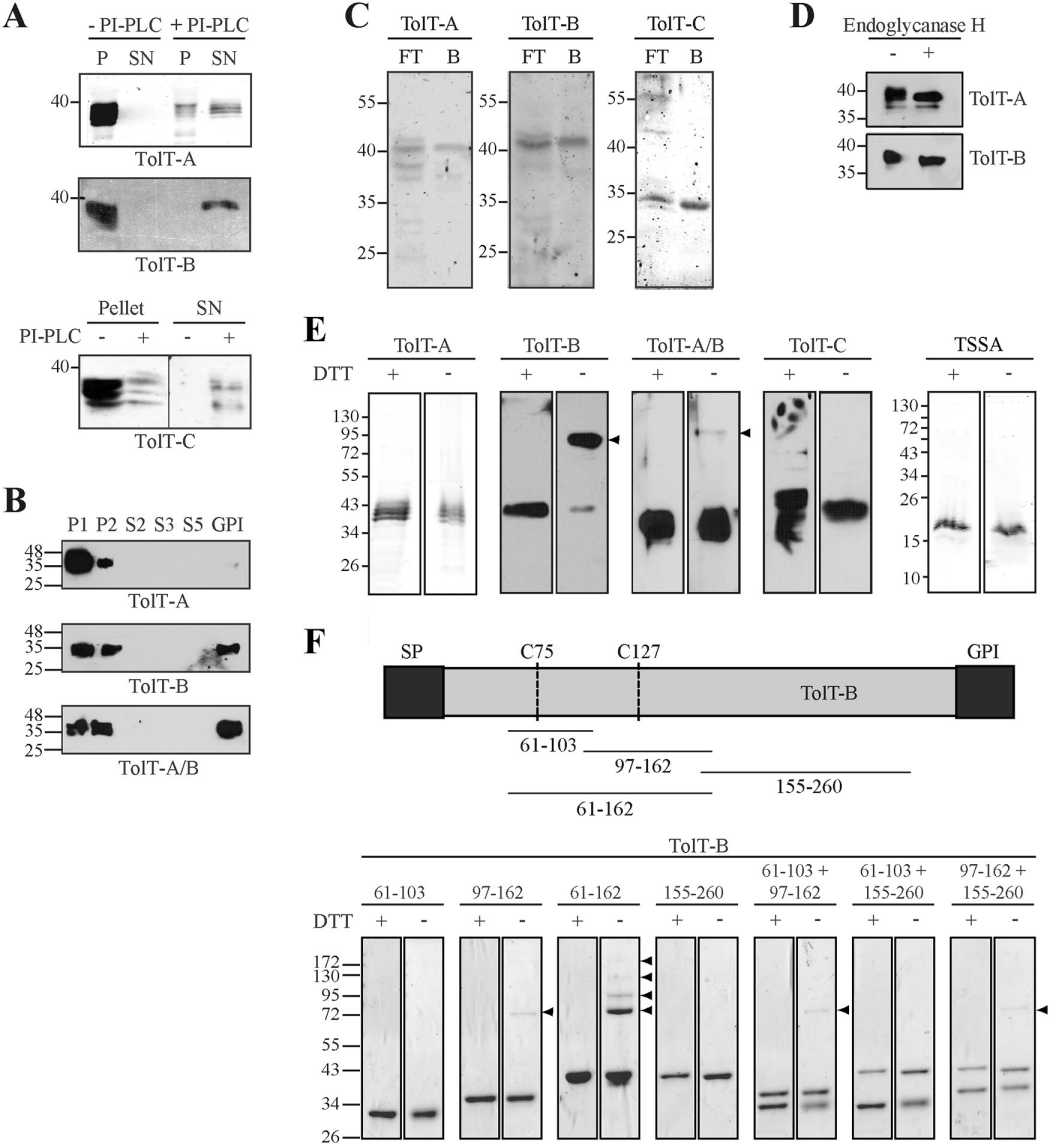
Biochemical features of TolT products. **A)** Intact trypomastigotes were treated (+) or not (-) with *B*. *cereus* PI-PLC for 30 min. Parasites were centrifuged, and aliquots from supernatants (SN) and total parasite lysates (P) were probed with the indicated antiserum. **B)** Trypomastigotes were fractionated with Triton X-114 and samples from each purification step (see Materials and methods) were probed with the indicated antiserum. **C)** Trypomastigote extracts were fractionated onto ConA-sepharose and aliquots of both flow-through (FT) and bound (B) fractions were probed with the indicated antiserum. **D)** Samples from trypomastigote extracts were treated (+) or not (-) with endoglycosidase H and probed with the indicated antiserum. **E)** Samples from trypomastigote extracts were resolved by SDS-PAGE under reducing (DTT +) and non-reducing (DTT -) conditions and probed with the indicated antiserum. **F)** Schematic illustration of a ‘canonical’ TolT-B protein showing the predicted signal peptide (SP), the GPI-anchoring signal (GPI) and the conserved cysteine residues (C75 and C127). Sequences derived from this protein were expressed as GST-fusion molecules and the residues spanned by each construct are shown below (numbers indicate amino acid positions relative to the initial methionine). Samples (3 μg) from the indicated GST-TolT-B fusion proteins were resolved by SDS-PAGE under reducing (DTT +) and non-reducing (DTT -) conditions and stained by Coomasie Brilliant Blue. In E) and F), arrowheads indicate the position of oligomeric species. Molecular markers (in kDa) are indicated.

To evaluate *in silico* predictions (Fig 3A), intact CL Brener trypomastigotes were firstly treated with PI-PLC, which specifically cleaves *T*. *cruzi* GPI anchors, and the supernatant and pellet fractions were analyzed by Western blot. As shown in Fig 6A, addition of PI-PLC caused the disappearance of TolT-A-, TolT-B- and TolT-C-reactive bands from the parasite pellets, and their concomitant appearance in the supernatant fractions. Interestingly, a minor fraction of TolT-A and TolT-C, but not TolT-B molecules could not be solubilized by PI-PLC (Fig 6A). These PI-PLC-resistant species may bear a different kind of acyl group or, alternatively, they may correspond to immature molecules that have not yet reached the parasite surface.

We next purified total GPI-anchored proteins from CL Brener trypomastigotes taking advantage of their preferential fractionation in Triton X-114. Aliquots corresponding to the different fractions were analyzed by Western blot. As shown in Fig 6B, TolT-A and TolT-B species were found in P1 (total parasite lysates), P2 (containing mostly membrane-associated molecules excluded from GPI- and sterol-rich micro-domains), and GPI fractions (containing mostly GPI-anchored proteins). Together with PI-PLC data, these findings confirmed that TolT molecules (at least a major fraction of them) are anchored to the trypomastigote plasma membrane through a GPI lipid motif. The finding of TolT-A and TolT-C PI-PLC-resistant species (Fig 6A), and of inter-group variations in the GPI/P2 abundance ratio (Fig 6B) unveiled certain heterogeneity in the way TolT molecules become associated to the parasite surface, and suggest that a minor fraction of them, particularly from TolT-A and TolT-C groups, use a different acyl group to achieve this issue.

We also analyzed whether the consensus *N-*glycosylation sites predicted in the deduced TolT products (Fig 3A) had an attached oligosaccharide *in vivo*. To that end, we initially carried out Western blot assays of CL Brener trypomastigote lysates upon fractionation on ConA lectin. As shown in Fig 6C, part of TolT-A-, TolT-B and TolT-C-reactive products were recovered in the ConA-bound fractions, indicating that at least a fraction of them indeed bear high-mannose type glycans. Trypomastigote lysates were next treated with endoglycosidase H. In line with original ‘TolT’ results [9], this treatment increased the electrophoretic mobility of TolT-A and TolT-B molecules (Fig 6D). Interestingly, the lower TolT-A-reactive band was not affected by endoglycosidase H treatment, suggesting that it may correspond to non-glycosylated species. Together with ConA-fractionation data, these findings suggest that at least part of the species from each TolT group undergo *N-*glycosylation *in vivo*.

To explore a possible structural role of Cys residues on the mature region of TolT molecules (Fig 3A), trypomastigote extracts were resolved in parallel on reducing and non-reducing SDS-PAGE and evaluated by Western blot. As shown, TolT-B molecules appeared to assemble into oligomers, which translated into the appearance of ~100 kDa species in non-reducing SDS-PAGE (Fig 6E). Considering the apparent molecular mass of TolT-B monomers (~35 kDa), the ~100 kDa species may likely correspond to trimers. In sharp contrast, solely monomeric species were observed for TolT-A and TolT-C molecules under non-reducing conditions (Fig 6E). TSSA, a well-characterized GPI-anchored molecule from the trypomastigote surface and devoid of Cys residues on its mature region [35,41] was used as control for these assays, and yielded solely monomeric species (Fig 6E). As expected, the ~100 kDa band was also revealed by the TolTA/B antiserum (Fig 6E). However, and since this antiserum recognized both TolTA (exclusively monomeric) and TolT-B (mostly trimeric), the ratio between trimers/monomers was shifted towards monomers (Fig 6E).

When expressed in a bacterial system, a TolT-B fusion molecule bearing both Cys residues 75 and 127 (GST-TolT-B 61–162) was able to assemble into multiple oligomeric forms (Fig 6F). GST-TolTB 97–162 (bearing solely Cys 127), on the other hand, yielded monomeric species and low amounts of a ~70 kDa species, likely a dimer, suggesting that the participation of both Cys residues is a pre-requisite in order to get trimeric and/or higher order aggregates. GST-TolT-B 61–103 (bearing solely Cys 75) was not able to dimerize under non-reducing conditions (Fig 6F), similar to GST-TolT-B 155–260 (bearing no Cys residue) used as negative control. These findings indicated that Cys 75 residue in GST-TolT-B 61–103 cannot engage into disulfide bond formation, likely due to structural constraints. Accordingly, *in vitro* incubation of GST-TolT-B 97–162 alone or in combination with GST-TolT-B 61–103 yielded the same profiling of high molecular mass species, i.e. solely the ~70 kDa band corresponding to the dimeric form of GST-TolT-B 97–162 (Fig 6F). Together, these findings strongly suggest i) that TolT-B, but neither TolT-A nor TolT-C molecules, are spontaneously assembled into trimers *in vivo*, on the surface of the trypomastigote; and ii) that trimeric TolT-B species are sustained by covalent inter-molecular disulfide bonds involving both Cys 127 and Cys 75.

### TolT antibody recognition is focused to the mature, C-terminal regions of TolT-A and TolT-B members

TolT molecules were shown to elicit B-cell responses during *T*. *cruzi* infection in humans, and 3 full-length variants (2 ‘canonical’ TolT-B and 1 TolT-A member) were thereby included in a 16-recombinant protein-based, multiplexed assay for serodiagnosis of Chagas disease [42]. However, neither the fine antigenic structure of TolT molecules nor the impact of herein evidenced diversity on the TolT epitopic landscape was yet addressed. Three TolT-A (TcCLB.506617.10, TcCLB.504157.130 and TcCLB.508767.20), 2 ‘canonical’ TolT-B (TcCLB.510433.20 and TcCLB.504277.11), 1 ‘GPI-less’ TolT-B (TcCLB.504277.20), and 1 TolT-C (TcCLB.504277.30) sequences were firstly analyzed using high-density peptide microarrays [37]. Briefly, overlapping sequences with 1-amino acid residue offset were probed with IgG samples from different pools of chronic Chagasic sera. Arrays were processed firstly with normal human IgG to assess the background reactivity, and final antigenic profiles calculated by subtraction [37].

**Fig 7:**
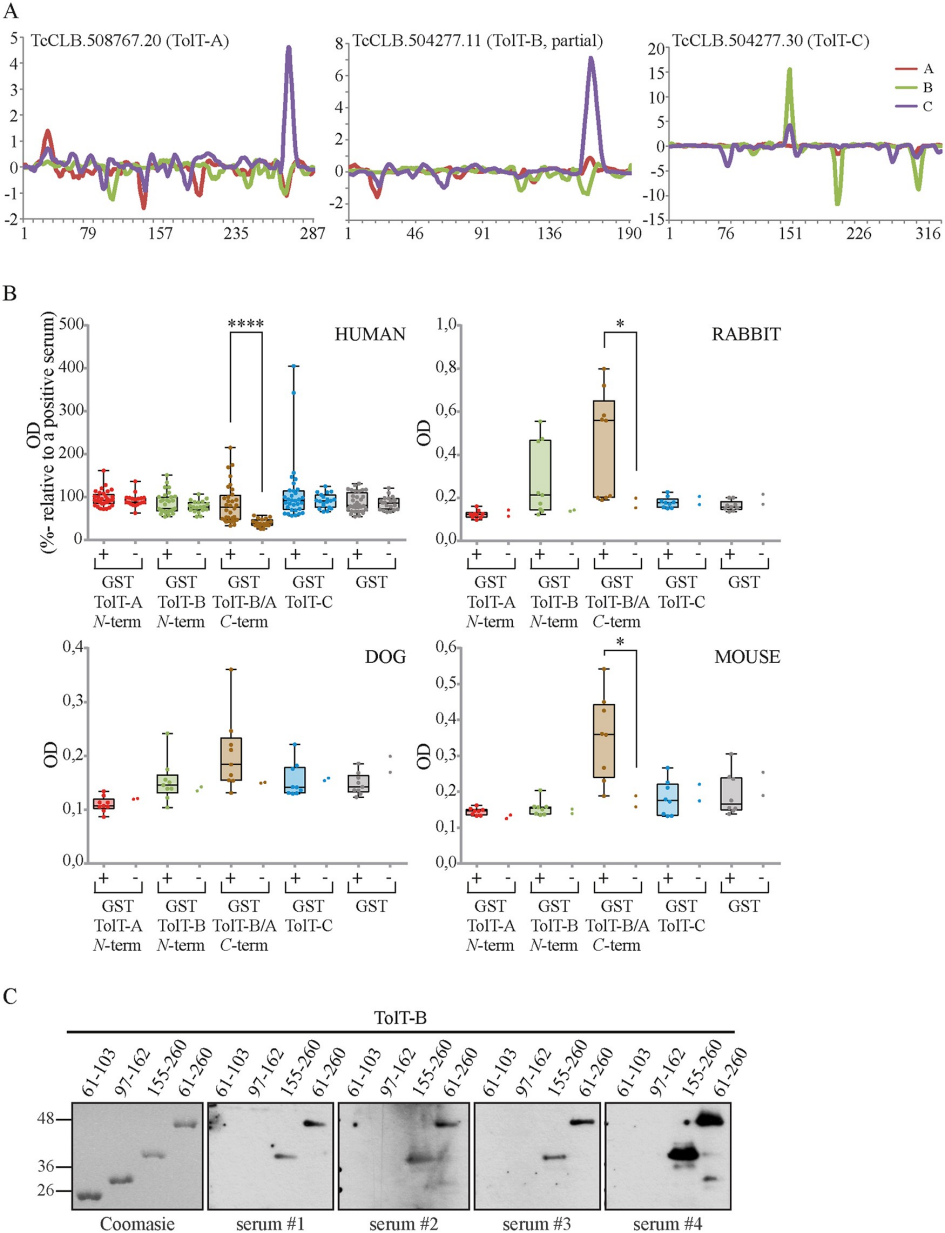
Antibody binding to TolT peptides is focused to the conserved, mature *C*-terminal region of TolT-A and TolT-B products. **A)** Microarrays composed of 15mer peptides overlapping by 14 residues spanning the deduced sequence of TolT members were probed, in duplicate, with three IgG samples purified from different pools of chronic Chagasic sera (denoted as A, B and C). The mean reactivity from each IgG sample toward every peptide (in arbitrary units of fluorescence) vs amino acid position (taking as residue 1 the predicted initial Meth residue, except for TcCLB.504277.11) is indicated. Solely those TolT molecules yielding positive results in at least one assay (see text and ref [37] for details) are shown. **B)** Dot plot analysis of ELISA results using different GST-TolT fusion proteins: TolT-A F54-T174 (GST-TolT-A *N*-term), TolT-B Q61-L162 (GST-TolT-B *N*-term), TolT-B G155-R260 (GST-TolT-B/A *C*-term), TolT-C A83 to D313 (GST-TolT-C) and GST. The sequences that were expressed as GST-fusion molecules and the residues spanned by each construct (numbers indicate amino acid positions relative to the initial methionine) are indicated. The ELISA plates were coated with the indicated antigen and incubated with 31 serum samples from chronic Chagas-positive individuals (+) or 19 non-infected individuals (-). *T*. *cruzi*-infected dogs (*n* = 9), rabbits (*n* = 9) and mice (*n* = 8) were also tested along with 2 non-infected individuals from the same species. The median and SD for each group are indicated by box and whiskers. Asterisks denote significant differences between the population medians (*P* < 0.0001 for humans and *P* < 0.05 for mice and rabbits, Mann-Whitney test). **C)** Aliquots (1 μg) of the indicated GST-TolT-B fusion proteins were stained by Coomasie Brilliant blue (left panel) or probed with 4 different chronic Chagasic sera by Western blotting.

Oddly, TolT products displayed an overall very low reactivity when assessed by this approach. As shown in Fig 7A, solely TcCLB.504277.30 (TolT-C), TcCLB.504277.11 (‘canonical’ TolT-B) and TcCLB.508767.20 (TolT-A) could be annotated as ‘weak antigens’ in the context of the whole array (see also [37]). Moreover, each one of them actually displayed a single ‘antigenic peak’, i.e. a stretch of consecutive peptides yielding reactivities above the established cutoff (Fig 7A). The TolT-C antigenic peak encompassed the sequence ^145^AAVDADTAALAALLEVLQ, and was recognized by 2 out of 3 analyzed pools of sera (Fig 7A). In addition, TolT-C yielded a couple of negative antigenic peaks in one assay, suggesting that these sequences may be recognized by IgGs from healthy individuals (Fig 7A) [37]. TolT-A and TolT-B weak antigenic peaks encompassed the same sequence (TATRIQRTRPRVD), located on their *C-*terminal region, which was recognized by solely 1 analyzed pool of sera (Fig 7A). Large variations in the length of TcCLB.504277.11 and TcCLB.508767.20 deduced proteins, and hence in the relative location of the TATRIQRTRPRVD epitope within them, reflected that TcCLB.504277.11 was annotated as an *N*-terminal truncated protein (see Fig 2).

The antigenic profile of TolT proteins was next evaluated using recombinant proteins. To that end, a series of GST-fusion molecules were generated and purified from engineered bacteria. These molecules were used in ELISA tests to search for specific antibodies in serum samples from chronic Chagasic patients. As shown in Fig 7B, TolT-specific antibodies were indeed detected by this procedure in a fraction of assayed Chagasic sera and in none of the 19 normal serum samples (Fig 7B). Every TolT-positive serum recognized the *C*-terminal region conserved among TolT-A and TolT-B molecules (GST-TolT-B/A *C*-term protein, Fig 7B). Due to cloning/expression purposes, the TATRIQRTRPRVD sequence highlighted on the microarray assays (Fig 7A) was not included in the GST-TolT-B/A *C*-term protein, hence indicating the presence of additional B-cell epitope(s) in this molecule. In addition to the GST-TolT-B/A *C*-term protein, solely one sample reacted against the *N*-terminal region of TolT-A and two samples against TolT-C (Fig 7B). Western blot assays to a panel of TolT-B deletion mutants further stressed the significantly skewed recognition profile of anti-TolT antibodies elicited during *T*. *cruzi* infection in humans. As shown in Fig 7C, the recognition of four TolT-reactive Chagasic sera not included in our ELISA panel was also restricted to the conserved *C*-terminal region (residues 155–260, according to TolT-B). Quite similar results were obtained upon testing serum samples from *T*. *cruzi*-infected mice, rabbits and dogs by ELISA (Fig 7B).

**Fig 8:**
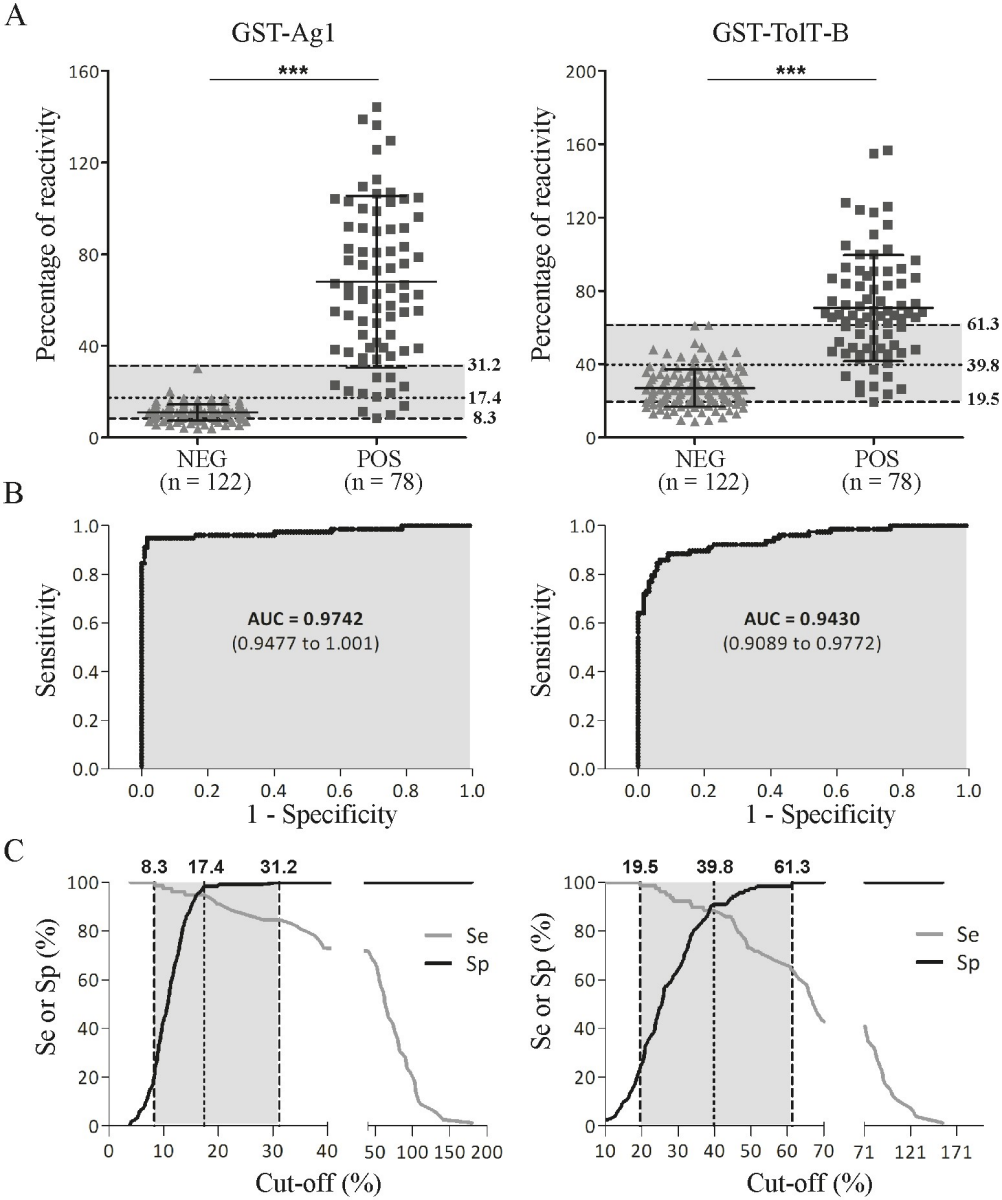
TolT-B constitutes an appealing candidate for Chagas disease serodiagnosis. **A)** Dot plot analysis. Positive (POS) and negative (NEG) serum samples were tested with the fluorescence magnetic beads immunoassay using microbeads functionalized with GST-Ag1 or GST-TolT-B Q61 to R260 proteins (see Materials and methods for details). The mean and standard deviation for each group are indicated (GST-Ag1: POS, 68.0 ± 37.5; NEG, 10.9 ± 3.6. GST-TolT-B: POS, 70.7 ± 28.9; NEG, 27.0 ± 10.2). ***, *P* < 0.0001; Mann-Whitney test. **B)** ROC curve analyses were carried out using as reference samples the POS and NEG groups included in the dotplot analyses. AUC, area under the ROC curve; values in parentheses indicate the 95% confidence interval. **C)** TG-ROC plot of the results. The dashed lines indicate the cut-off values for which maximal diagnostic sensitivity (Se) or specificity (Sp) were achieved (8.3 and 31.2% for Ag1, and 19.5 and 61.3 for TolT). These two cut-off values represent the bounds of an intermediate range of reactivity values (shaded areas). The dotted lines indicate the cut-off value that concurrently optimizes the Se and Sp (17.4% for Ag1 and 39.8% for TolT).

The diagnostic performance of a GST-TolT-B fusion protein spanning most of its mature region (residues Q61 to R260, S2 Table) was evaluated by a recently developed FMBIA test, using 2 panels of serum samples obtained from non-infected individuals (*n =* 122) or from patients with chronic Chagas disease (*n =* 78). The latter was heterogeneous, and included people living and/or raised in different endemic areas from Argentina or neighbor countries, and hence most probably parasitized by different *T*. *cruzi* strains [43]. For comparison purposes, the same analysis was performed in parallel using GST-Ag1, a well-established Chagas disease serodiagnostic reagent [27]. For both antigens, a significant difference in the overall reactivity values between the negative and positive populations was obtained (*P* < 0.0001; Fig 8A). Most importanly, the area under the ROC curve for GST-TolT-B showed that this is a highly performant diagnostic classifier, with an area under the curve (AUC) value very similar to that of GST-Ag1 (0.9430; 95% CI, 0.9089–0.9772 for GST-TolT-B and 0.9742, 95% CI, 0.9477–1 for GST-Ag1) (Fig 8B). Plots of the diagnostic sensitivity and specificity of these assays as a function of the cut-off values (TG-ROCplot) indicated a cut-off value that concurrently optimizes both parameters of 17.4% for Ag1 and of 39.8% for GST-TolT-B. Overall, these results indicate that a recombinant, GST-fusion protein spanning most of the mature region of a ‘canonical’ TolT-B molecule, including its antigenic and conserved *C*-terminus, provides an appealing reagent for Chagas disease serodiagnosis.

## Discussion

Despite original proposals [9,11], we herein show that TolT constitutes a complex family of genes in kinetoplastids. According to phylogenomic data, it is apparently restricted to the branch of stercorarian trypanosomes, i.e. trypanosomes that develop in the hindgut of the insect vector, such as *T*. *cruzi*, *T*. *cruzi marinkellei*, *T*. *grayi* and *T*. *rangeli*. Even though *T*. *rangeli* is also able to develop in the triatomine salivary glands, taxonomic studies indicated it failed to group with other strict salivarian trypanosomes [44]. Solely in the *T*. *cruzi* CL Brener reference clone, we were able to find 12 *TolT* genes distributed in two chromosome clusters. Moreover, comparative analyses carried out in TCC, a closely related strain with a better-quality genome assembly and annotation [26], strongly suggest that such TolT gene dosage may be an underestimation. *T*. *cruzi* TolT genomic clusters comprise a discrete number of tandemly arranged genes from the same group, i.e. TolT-A genes in chromosome 23 cluster and TolT-B genes in chromosome 35 cluster. Within each cluster, TolT genes show minor polymorphisms among them. As extensively discussed, gene expansion by tandem duplication without further differentiation likely constitutes a kinetoplastid evolutionary strategy to increase protein yield in the absence of transcriptional regulation [45]. Indeed, mRNA and protein expression data roughly correlate with the estimated gene dosage for each TolT group. Interestingly, an additional and ‘different’ TolT gene is found immediately downstream of both TolT clusters, strongly suggesting that it evolved by mutation accumulation on a previously duplicated copy from another group. In the case of TolT-C genes, which display rather low similarity to the remaining *T*. *cruzi TolT* genes (~47% identity to any *TolT-A* gene), this particular genomic disposition was one pivotal criterion for their inclusion within the TolT family.

Overall, the most parsimonious hypothesis integrating these findings suggests that *TolT* emerged in an ancestor from trypanosomes, early after the divergence of the salivarian branch [46]. The acquisition of its embedded bacterial TolA-like motif may have occurred either by horizontal gene transfer, as previously proposed for other trypanosomatid molecules [47,48] or by convergent evolution. Whatever the case, the original TolT sequence likely underwent subsequent events of gene duplication followed (or not) by diversification (and eventually pseudogenization), thus leading to the formation of a rather complex family of genes. A rather similar evolutionary path, characterized by remarkable expansion and diversification, seems to have been followed by several *T*. *cruzi* gene families coding for surface molecules involved in the interaction with the mammalian and/or vector hosts [49].

*TolT* deduced proteins show a biased amino acid composition and molecular signatures of surface localization and/or secretion such as cleavable SP, glycosylation, and lipid modification. Based on topology predictions and experimental data, it could be inferred that, upon maturation in the secretory pathway, TolT molecules become tethered to the outer leaflet of the flagellar membrane *via* their *C-*termini. TolT membrane anchor most likely occurs post-translationally, by the addition of a GPI lipid moiety early upon their entry to the secretory pathway [50]. However, and as mentioned, our data is compatible with the possibility that a minor fraction of them, particularly those from TolT-A and TolT-C groups, use an alternative acyl group (i.e. palmitoyl) to achieve this issue. The only exception to the topological model proposed above would be TcCLB.504277.20, which loses its predicted GPI-anchoring signal due to focalized mutation accumulation. As shown for *T*. *cruzi* surface mucins, recombinant GPI-less variants (i.e. deletion mutants lacking the GPI-anchoring signal) accumulate in the endoplasmic reticulum, with only a minor fraction being ultimately released to the medium as anchorless products [51]. Further studies, currently underway, will be required to address TcCLB.504277.20 biochemical properties and sub-cellular distribution.

A unique and unifying feature of TolT molecules is that they bear similarity to the central region of TolA proteins. These are integral membrane molecules dedicated to maintain outer membrane stability in different bacteria such as *Escherichia coli* and *Pseudomonas aeruginosa* [10,52,53], being used also for the uptake of several filamentous phages and bacterial toxins called colicins [54]. TolA molecules are anchored to the inner bacterial membrane *via* their *N*-terminal domain, and display a central region made up essentially of alanine-rich stretches that show very stable helix conformation. Importantly, the TolA alanine-rich central region seems to play mainly a structural role, allowing the projection of the functional *C*-terminal region across the bacterial periplasm [54]. Though not experimentally proven, different algorithms predict that the TolA-like motif present in *T*. *cruzi* TolT molecules also encompasses a very long and unique αhelix, which likely adopts a rod-like structure on the trypomastigote membrane. In such a case, the acquisition of the TolA-like motif on the TolT central region may have been selected for as a crafty strategy to ensure the protrusion, and hence maximize the exposition, of the outermost (and variable) mature *N*-terminal region. Variations on this theme, leading to the projection of functional domains across the parasite glycocalix have been proposed for other *T*. *cruzi* surface molecules [8,55,56]. Most importantly, the overall topological model predicts a functional role for the TolT mature *N*-terminal regions; which according to their sub-cellular distribution may be related to the interaction between the flagellum and the trypomastigote body [57].

Original IIF assays carried out by Saborio et al showed that ‘TolT’ (presumably a TolT-A molecule according to our current classification) localized to the trypomastigote flagellum surface, apparently in the part of this structure in contact with the parasite body [9]. Here we assessed this sub-cellular localization for every TolT product, suggesting that despite their amino acid and biochemical variations all of them share the targeting signals responsible for this selective trafficking. Most interestingly, TolT-A and TolT-B products distribute in discrete foci along the surface of the trypomastigote flagellum. This is consistent with recent finding showing a biased lipid composition for the flagellar membrane of trypanosomatids, which apparently promotes the accumulation of GPI- and other kinds of acyl-anchored proteins into lipid-raft-like structures [58]. Moreover, the punctuate and non-overlapping pattern observed for TolT-A and TolT-B molecules builds upon our hypothesis of the trypomastigote membrane as a highly organized structure made up of multiple and discrete nanoscale domains bearing different protein composition [8,14]. Inter-molecular disulfide bonds leading to the formation of TolT-B homopolymers may also contribute to the formation/organization of these particular domains. Alternatively, disulfide bonds may have a rather classical structural role as a TolT-B quality control system in the endoplasmic reticulum [59] or, as shown for other protozoan surface antigens, in the undermining of the mammalian host immune response [60]. In this sense, it should be emphasized the lack of antibody response to TolT *N-*terminal regions. Our discoveries also raise the interesting possibility that the state of extracellular reduction-oxidation reactions on the vicinity of the trypomastigote flagellum may regulate the polymerization status of TolT-B molecules *in vivo*, which in turn may affect their yet-to-be-addressed functional and/or signaling properties [61–63]. *In vivo* studies using site-specific mutants and defined conditions should help to clarify these issues.

In addition to trypomastigotes, TolT products are also expressed on amastigote forms. Most interestingly, amastigote-expressed molecules accumulate on intracellular compartments. TolT-A (and TolT-C) molecules accumulate in discrete regions towards the posterior end of the amastigote, which may correspond to degradative organelles described in *T*. *cruzi* insect-dwelling forms [64]. TolT-B (and TolT-C) molecules, on the other hand, are likely retained in the flagellar pocket, the organelle that contributes to the traffic of GPI-anchored proteins between the Golgi complex and the plasma membrane [65] whereas TolT-C molecules accumulate in an undefined compartment juxtaposed to the kDNA. Whether these intracellularly-displayed molecules correspond to immature proteins *en route* to the amastigote membrane and/or to recycled species targeted for degradation remains to be addressed.

Our immunological characterizations support TolT molecules as targets of the immune response during *T*. *cruzi* infections. Indeed, by using a recently developed FMBIA test we show that a recombinant, GST-fusion protein spanning most of the mature region of a ‘canonical’ TolT-B molecule exhibits quite similar diagnostic performance than a well-established *T*. *cruzi* antigen, included in commercial serodiagnostic tests [27]. Antibody recognition seems to be focused towards peptides from the TolT-A/TolT-B conserved *C*-terminus, independently of the evaluated mammalian species. These findings may be attributed to intrinsic antigenic issues, i.e. biased distribution of B-cell epitopes, to the over-representation (in molar terms) of the TolT conserved *C-*terminal region or to the *in vivo* molecular shielding of TolT *N*-terminal regions by structural constraints and/or post-translational modifications. The latter hypothesis is however not consistent with IIF-based data showing that these regions are readily available to antibodies on both trypomastigote and amastigote forms. The lack of correlation between the peptide chip- and recombinant protein-based approaches, which is unique among other tested *T*. *cruzi* molecules [31,33,37], suggest that B-cell epitopes from Tol-T molecules are not strictly linear in nature.

In summary, we have shown that TolT constitutes a complex family of genes in *T*. *cruzi*, which could be split into three robust groups displaying differences in their structure, sub-cellular distribution, post-translational modification and antigenic composition. The fact that these molecules i) are abundantly expressed on *T*. *cruzi* developmental stages that dwell in the mammalian host; ii) provide robust and reliable reagents for the improvement/development of novel diagnostic and/or epidemiologic applications (see also [42]); and iii) were shown to constitute appealing vaccine candidates [11] indicate that they constitute excellent targets for molecular intervention in Chagas disease.

## Supporting information

S1 Table Oligonucleotides used in this study. (DOC)

S2 Table Antisera used in this study. (DOC)

S1 Fig *ClustalW* alignment of *T*. *cruzi* CL Brener *TolT-A* genes. The translation initiation ATG codon is boxed and the position of the apparent single nucleotide deletion in TcCLB.511109.10 is indicated with an asterisk.
(TIF)

S2 Fig *ClustalW* alignment of the *C*-terminal region of selected *TolT-B* deduced products. The sequence of the predicted GPI-anchoring signal is shaded in red and the glycolipid acceptor residue (ɷ) is indicated.
(TIF)

S3 Fig Sequence and structure of the *T*. *cruzi* CL Brener TcCLB.506815.20 gene. Sequences corresponding to a partial *TolT-B* gene (green), a putative intergenic region (IGR, grey) and a complete *TolT-C* gene (light blue) found within TcCLB.506815.20 are schematically indicated.
(TIF)

S4 Fig *Clustal W* alignment of TolT products identified in *T*. *cruzi* CL Brener. (TIF)
