# Supplementary material for: Molecular and antigenic characterization of Trypanosoma cruzi TolT proteins
Source: PLoS Negl Trop Dis. 2019 Mar 14;13(3):e0007245. doi: 10.1371/journal.pntd.0007245 (PMC6435186; doi:10.1371/journal.pntd.0007245)
Supplement: S1 Table — (DOC) [file pntd.0007245.s001.doc]

**Supplementary Table 1** – Oligonucleotides used in this study.

| **Name^1^** | **Description^2^** | **Sequence (5’ to 3’)** |
| --- | --- | --- |
| A RT_Fw_  A RT_Rv_ | TolT-A  506617.10/506617.20 | CTGGGCTTGAGAAAGAAGCAG  CTGGAATTCCTGCTCCTCCT |
| B RT_Fw_  B RT_Rv_ | TolT-B  508767.10/510433.20 | AGGCTACAAGGATGAGCAGG  CCAACCTTAGTCTTCTCGTCC |
| C RT_Fw_  C RT_Rv_ | TolT-C  504277.30 | CCAAGAAATACGCCGAAGAG  TTTGTCAGTCGAATCTGCAGAG |
| Calm_Fw_  Calm_Rv_ | TcCalmodulin  506391.10/506391.20/  507483.30/507483.39/  507483.50 | CCCGACGGAGGCGGAGCTGC  GTCCACGTCGGCCTCGCGGA |
| GAPDH_Fw_  GAPDH_Rv_ | TcGAPDH  506943.50/506943.60/  509065.60/509065.70 | GTGCGGCTGCTGTCAACA  AAAGACATGCCCGTCAGCTT |
| Oligo-dT-anchor |  | GCGAGCTCCGCGGCCGCGTTTTTTTTTTTTTTTTTT |
| A_Fw_  A_Rv_ | TolT-A F54 to T174^2^ | GCggatccTTTGACTGGGCATTCAAG^3^  GActcgagCTAAGTATGATTTGCCGCCTT |
| B_Fw_1  B_Rv_1 | TolT-B Q61 to S103 | CGggatccCAGGAGTACGCTGATGAGGCT  CGgaattcGCTCATCCTTGTAGCCTCAGA |
| B_Fw_2  B_Rv_2 | TolT-B S97 to L162 | CGggatccTCTGAGGCTACAAGGATGAGC  CGgaattcCAACAGCTCCGGTCCACTTCC |
| B_Fw_3  B_Rv_3 | TolT-B G155 to A260 | CGggatccGGAAGTGGACCGGAGCTGTTG  CGgaattcGCGCTGCGTCATTCCCCTTCT |
| C_Fw_  C_Rv_ | TolT-C A83 to D313 | GAggatccGCGACAATGCAATGCATG  GActcgagCTAACTCCCACTGCGTCTGACTC |
| pGEX_Fw_^4^  pGEX_Rv_ |  | GGGCTGGCAAGCCACGTTTGGT  CCGGGAGCTGCATGTGTCAGA |
| T7^4^  SP6 |  | AATACGACTCACTATAGGG  ATTTAGGTGACACTATAG |

^1^ Oligonucleotides from the upper part of the table were used for Real-Time qPCR quantitation whereas those from the bottom part were used for cloning procedures.

^2^Accession numbers for *T. cruzi* CL Brener genes are provided as: TcCLB.XXXXXX.XX.

^2^The residues spanned by each deduced amplicon (numbers indicate amino acid positions relative to the initial methionine) are indicated.

^3^Restriction sites are underlined.

^4^Universal oligonucleotides.
