# Supplementary material for: Molecular and antigenic characterization of Trypanosoma cruzi TolT proteins
Source: PLoS Negl Trop Dis. 2019 Mar 14;13(3):e0007245. doi: 10.1371/journal.pntd.0007245 (PMC6435186; doi:10.1371/journal.pntd.0007245)
Supplement: S2 Table — (DOC) [file pntd.0007245.s002.doc]

**Supplementary Table 2** – Antisera used in this study.

| **Antiserum Label** | **Immunogen^1^** | **Species** |
| --- | --- | --- |
| TolT-A | Fragment F54-T174 of TcCLB.506617.10 | Mouse/Rat |
| TolT-B | Fragment Q61-S103 of TcCLB.510433.20 | Mouse |
| TolT-A/B | Fragment G155-R260 of TcCLB.510433.20 | Mouse |
| TolT-C | Fragment A83-D313 of TcCLB.504277.30 | Mouse |
|  |  |  |

^1^The residues spanned by each construct (numbers indicate amino acid positions relative to the initial methionine) are indicated. All of this constructs were expressed as GST-fusion proteins.
