# Supplementary figures and images for: Molecular and antigenic characterization of Trypanosoma cruzi TolT proteins
Source: PLoS Negl Trop Dis. 2019 Mar 14;13(3):e0007245. doi: 10.1371/journal.pntd.0007245 (PMC6435186; doi:10.1371/journal.pntd.0007245)

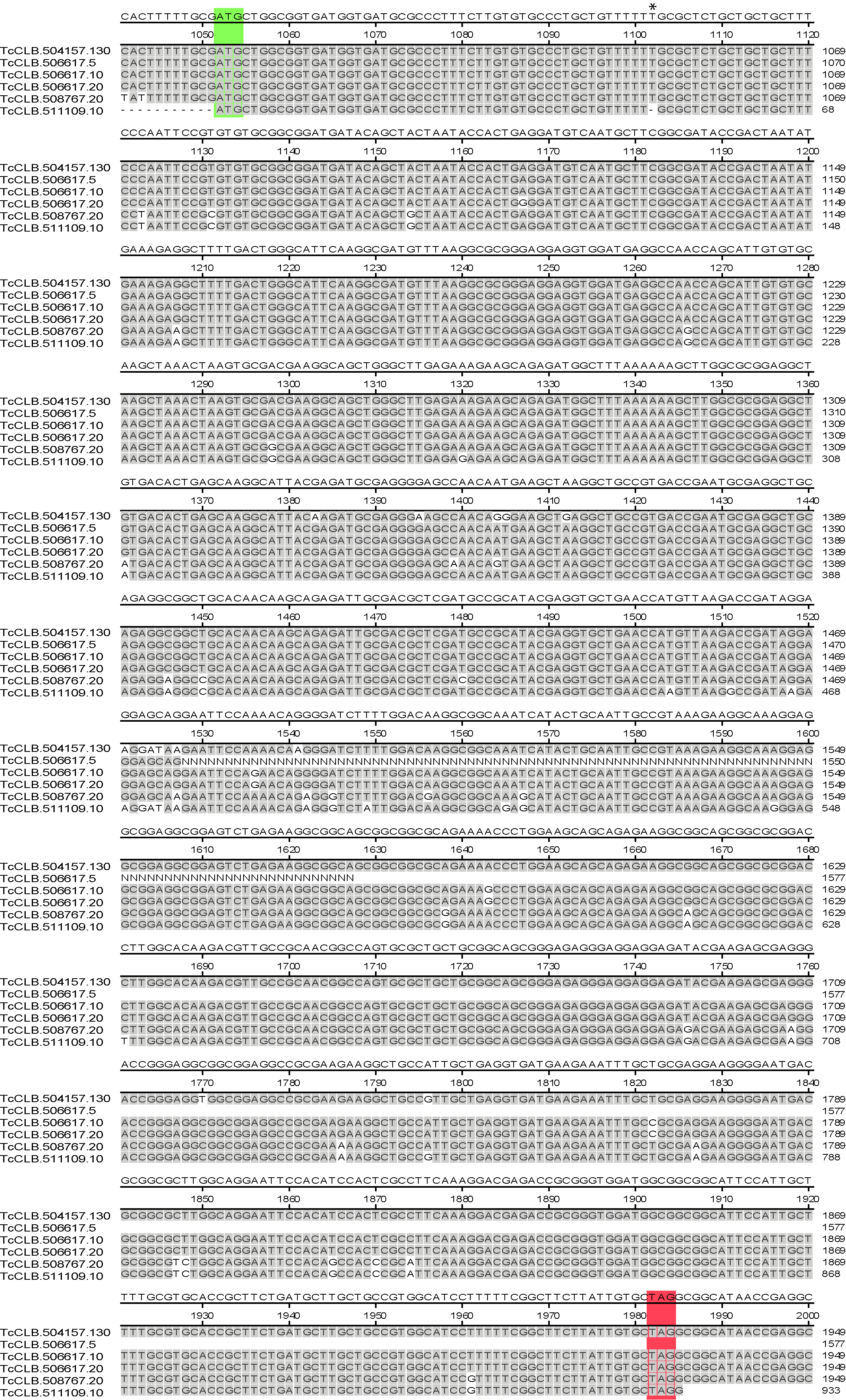

Supplement: S1 Fig — The translation initiation ATG codon is boxed and the position of the apparent single nucleotide deletion in TcCLB.511109.10 is indicated with an asterisk. (TIF) [file pntd.0007245.s003.tif]

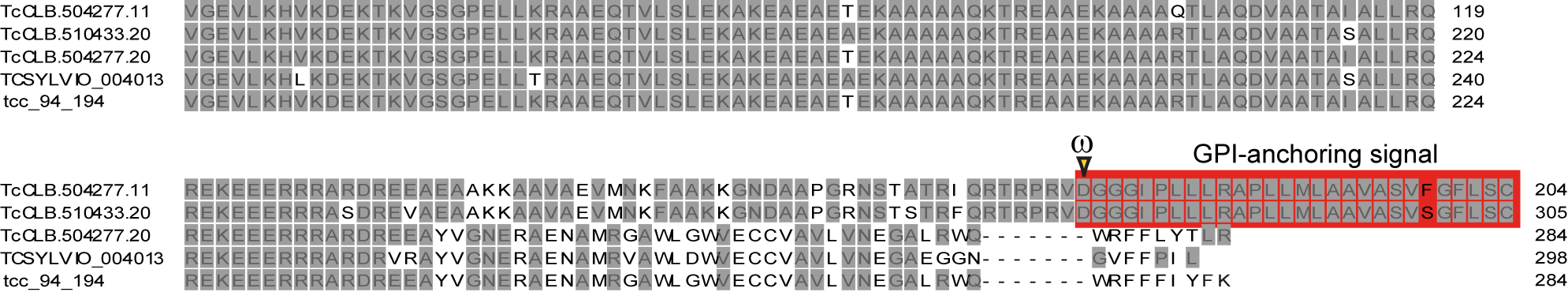

Supplement: S2 Fig — The sequence of the predicted GPI-anchoring signal is shaded in red and the glycolipid acceptor residue (ɷ) is indicated. (TIF) [file pntd.0007245.s004.tif]

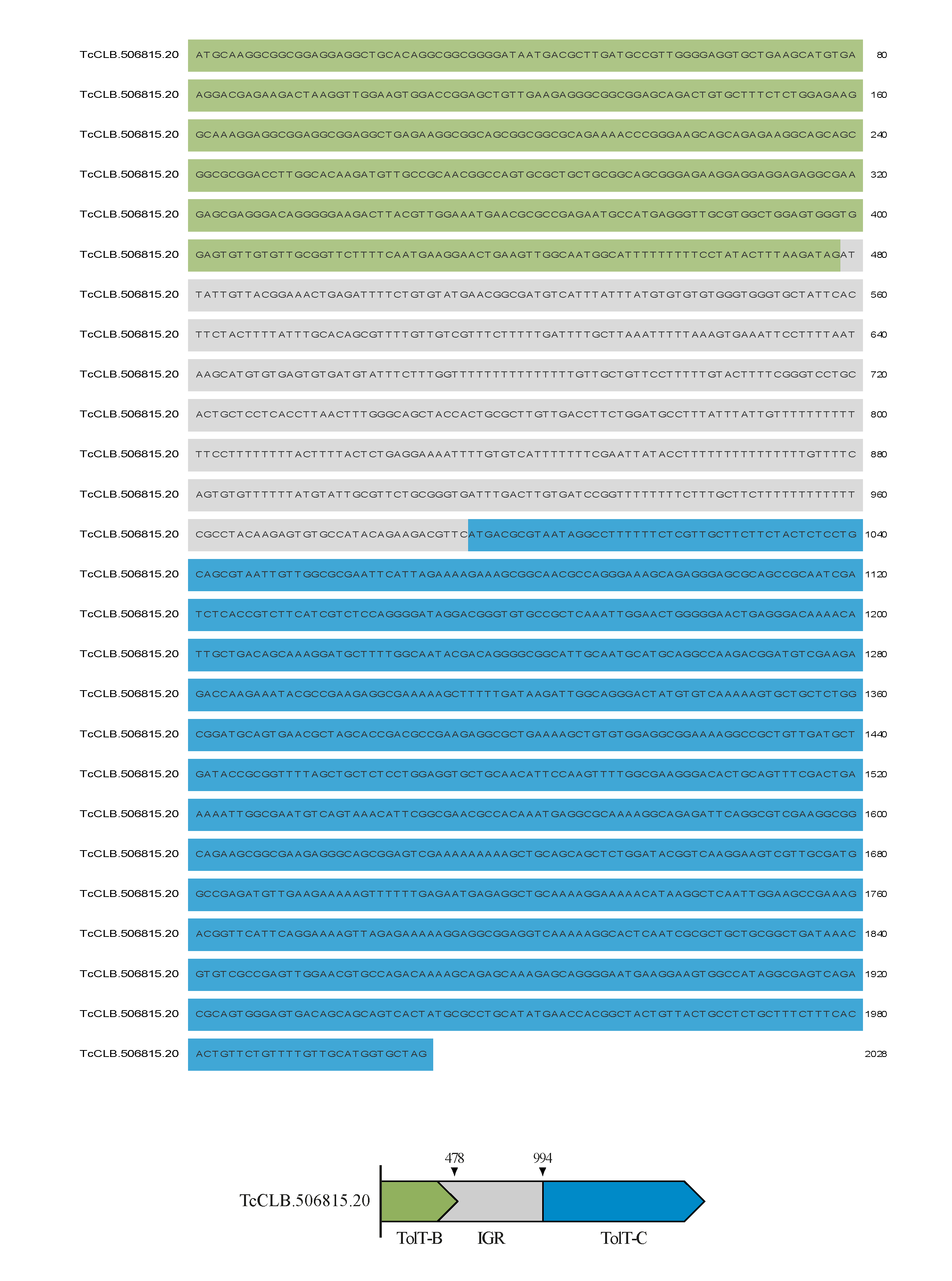

Supplement: S3 Fig — Sequences corresponding to a partial TolT-B gene (green), a putative intergenic region (IGR, grey) and a complete TolT-C gene (light blue) found within TcCLB.506815.20 are schematically indicated. (TIF) [file pntd.0007245.s005.tif]

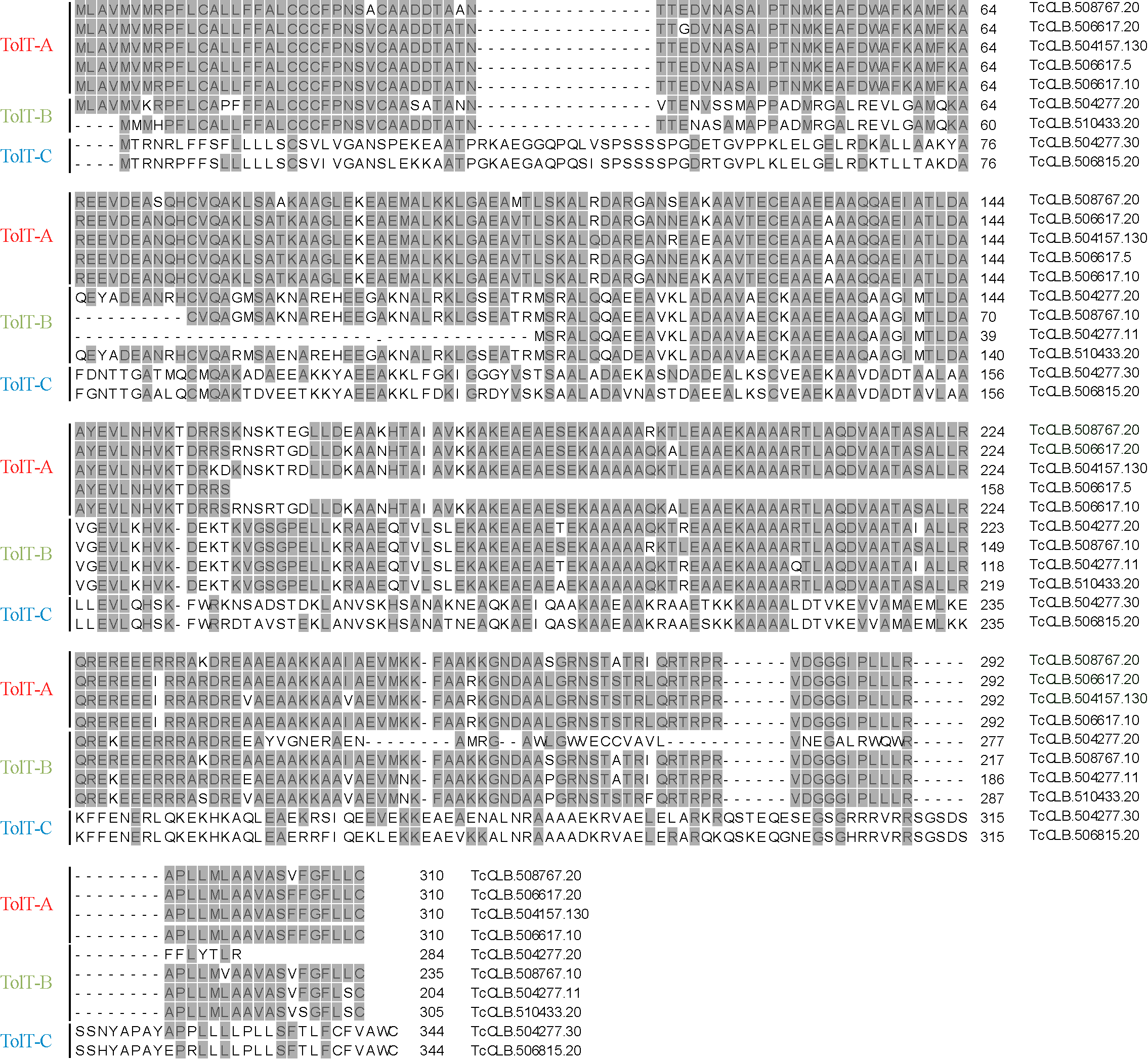

Supplement: S4 Fig — (TIF) [file pntd.0007245.s006.tif]
